# Strategies to reduce antimicrobials in livestock and aquaculture, and their impact under field conditions: a structured scoping literature review

**DOI:** 10.1093/jac/dkad350

**Published:** 2023-11-10

**Authors:** João Sucena Afonso, Mahmoud El Tholth, K Marie Mcintyre, Luís Pedro Carmo, Lucy Coyne, Diego Manriquez, Didier Raboisson, Guillaume Lhermie, Jonathan Rushton

**Affiliations:** Department of Livestock and One Health, Institute of Infection, Veterinary & Ecological Sciences, University of Liverpool, Liverpool, UK; Global Academy of Agriculture and Food Systems, The Royal (Dick) School of Veterinary Studies, The University of Edinburgh, Edinburgh, UK; Department of Health Studies, Royal Holloway University of London, Egham, UK; Hygiene and Preventive Medicine Department, Faculty of Veterinary Medicine, Kafrelsheikh University, Kafr el-sheikh, Egypt; Modelling, Evidence and Policy group, School of Natural and Environmental Sciences, Newcastle University, Newcastle upon Tyne, UK; Norwegian Veterinary Institute, Oslo, Norway; National Office of Animal Health, Stevenage, UK; CIRAD, UMR ASTRE, Montpellier, France, ASTRE, CIRAD, INRAE, University of Monpellier, Montpellier, Universite de Toulouse, ENVT, 31300, Toulouse, France; AgNext, Department of Animal Sciences, Colorado State University, Fort Collins, USA; CIRAD, UMR ASTRE, Montpellier, France, ASTRE, CIRAD, INRAE, University of Monpellier, Montpellier, Universite de Toulouse, ENVT, 31300, Toulouse, France; CIRAD, UMR ASTRE, Montpellier, France, ASTRE, CIRAD, INRAE, University of Monpellier, Montpellier, Universite de Toulouse, ENVT, 31300, Toulouse, France; Faculty of Veterinary Medicine, University of Calgary, Calgary, Canada; Department of Livestock and One Health, Institute of Infection, Veterinary & Ecological Sciences, University of Liverpool, Liverpool, UK

## Abstract

Antimicrobial resistance is a pandemic problem, causing substantial health and economic burdens. Antimicrobials are extensively used in livestock and aquaculture, exacerbating this global threat. Fostering the prudent use of antimicrobials will safeguard animal and human health. A lack of knowledge about alternatives to replace antimicrobials, and their effectiveness under field conditions, hampers changes in farming practices.

This work aimed to understand the impact of strategies to reduce antimicrobial usage (AMU) in livestock and aquaculture, under field conditions, using a structured scoping literature review. The Extension for Scoping Reviews of the Preferred Reporting Items for Systematic Reviews and Meta-Analysis guidelines (PRISMA-ScR) were followed and the Patient, Intervention, Comparison, Outcome, Time and Setting (PICOTS) framework used. Articles were identified from CAB Abstracts, MEDLINE and Scopus. A total of 7505 unique research articles were identified, 926 of which were eligible for full-text assessment; 203 articles were included in data extraction. Given heterogeneity across articles in the way alternatives to antimicrobials or interventions against their usage were described, there was a need to standardize these by grouping them in categories.

There were differences in the impacts of the strategies between and within species; this highlights the absence of a ‘one-size-fits-all’ solution. Nevertheless, some options seem more promising than others, as their impacts were consistently equivalent or positive when compared with animal performance using antimicrobials. This was particularly the case for bioactive protein and peptides, and feed/water management. The outcomes of this work provide data to inform cost-effectiveness assessments of strategies to reduce AMU.

## Introduction

Antimicrobial resistance (AMR) is a major global concern threatening both human and animal health, leading to increased mortality and morbidity, and resulting in severe economic losses. Predictive models have estimated that if no action is taken, by 2050 AMR will be responsible for the deaths of 10 million people per year, in addition to a 2.0%–3.5% reduction in global gross domestic production (between 60 and 100 trillion USD).^1^ Moreover, AMR impacts are felt unevenly within society, with increasing risks of situational and intergenerational poverty in vulnerable socioeconomic groups.^2^

AMR results from the natural adaptation of pathogens challenged with antimicrobials. Rates of AMR emergence, selection and consequent dissemination increase with antimicrobial usage (AMU), as pathogen exposure to antimicrobials provides opportunity for development of resistance.^3,4^ Antimicrobials are extensively used in livestock and aquaculture for growth promotion purposes and to manage the health of animals. Global AMU in 2020 was estimated at 99.5 thousand tonnes (95% CI 68.5–198.1 thousand tonnes), and is expected to have increased by 8% by 2030.^5^ There is limited evidence of the burden of AMR in humans that is attributable to livestock and aquaculture. Livestock production and aquaculture, however, inevitably play a role in global AMR threats due to present exposure levels of livestock and aquaculture species to antimicrobials, and our current knowledge of the pathways for development and transmission of AMR.^6,7^

In 2015, recognizing the human–animal–environment interface and anthropogenic nature of the AMR pandemic, a global action plan was developed using One Health approaches. The plan was endorsed by member countries of the WHO, highlighting the problem in global political agendas.^8^ It emphasizes the need to improve AMR awareness, conduct surveillance and research, reduce infection incidence through prevention strategies, foster the prudent use of antimicrobials, develop economic cases for sustainable investments to tackle AMR, and increase investment in the technological and scientific development of alternatives to current antimicrobials and interventions that aim to reduce AMU.^8^ More recently, the Quadripartite including the Food and Agriculture Organization of the United Nations (FAO), United Nations Environment Programme (UNEP), World Health Organization (WHO) and the World Organisation for Animal Health (WOAH), launched a new platform to facilitate stakeholder involvement, further strengthening the need for a global One Health effort to tackle AMR. In the livestock sector over the last decade, some country- and/or regional-level initiatives headed by both the public via government regulation, and the private sector by implementing standards, have successfully reduced AMU.^9^ Further reduction is required, whilst balancing the health and welfare of animals and the livelihoods of livestock and aquaculture producers.

A lack of understanding about strategies to replace AMU and their effectiveness under field conditions hampers change in farming practices. This knowledge gap is a critical hurdle to further reduce AMU in livestock and aquaculture production.^10^ Additionally, under a theory-of-change framework, producers will likely change their behaviour when believing that the benefits of reducing AMU exceed the costs.^11^ While advocating for sustainable reductions, it is important to provide evidence-based information on the application of alternatives to manage animal health and welfare.

The aims of this structured scoping literature review were to: (i) identify alternatives to antimicrobials and interventions to reduce their use in livestock and aquaculture under field production conditions; and (ii) qualitatively assess their impact on productivity (e.g. reduction in animal’s yield as a result of reducing AMU), economic (e.g. the costs of reducing stocking density in broiler production, or the cost efficiency of administering non-steroidal anti-inflammatory drugs in beef cattle for preventing bovine respiratory disease versus conventional use of antibiotics) or epidemiological outcomes (e.g. reduction in disease incidence as a result of implementing quarantine). Livestock included monogastric and ruminant livestock.

## Materials and methods

The objective of this literature review was to identify research articles reporting the impact of alternatives to antimicrobials or interventions to reduce AMU in livestock and aquaculture under field production conditions. The review included studies undertaken anywhere in the world in (non-experimental) production settings. Additionally, given the increasing relevance of the subject of anthelmintic resistance and following Purssell^12^ within this study, we have considered anthelmintics as within the antimicrobial group.

The structured scoping approach was developed by McIntyre *et al.* for the Global Burden of Animal Diseases (GBADs) programme, and based on the FAIR (findability, accessibility, interoperability and reusability) principles for good data management.^13,14^ An ‘inception table’ developed by the GBADs collaborators, and adapted from the Extension for Scoping Reviews of the Preferred Reporting Items for Systematic Reviews and Meta-Analysis guidelines (PRISMA-ScR) statement/checklist, was used to collate the information for the review protocol (e.g. review question, purpose, scope, search terms).^15^ This tool provided a structured framework, contextualizing the research question, developing search terms and optimizing the search strategy and code, making particular use of relevant peer-reviewed articles, grey literature and snowballing mechanisms (e.g. connectedpapers.com and the Ovid MEDLINE interface *Find similar* and *Find citing articles* functions) to improve searches and Medical Subject Headings (MeSH) terms utilized in the search strategy. The inception table is described in Table S1 (available as Supplementary data at *JAC* Online).

The PICOTS framework was followed to formulate the review question, define search terms and help formulate the search strategy; see Table 1.^16^

**Table 1.** Search terms developed for the structured scoping review using the adapted PICOTS protocol^16^

Adapted PICOTS field	Search terms/notes
Population	livestock, animal, production, food
Intervention	intervent*, strategy*, alternative*
Comparison	AMU, AMR, antimicrobial, usage, use, resistance
Outcome	econom*, impact*, effect*, benefit*, cost-effect*, cost-efficien*
Timing	Not restricted to any particular time period
Settings	Not restricted to any geography

Research articles were identified from three bibliographic databases: CAB Abstracts, Ovid MEDLINE and Scopus on 6 July 2022. Database-specific search strategies were developed and are described in Section 2 of the Supplementary data. All identified research articles were imported to Endnote X9 reference management software, and deduplication was conducted.^17^ The set of unique articles was exported (via Zotero) to SysRev (https://www.sysrev.com), a platform developed by Insilica, LLC for collaborative extraction of data from documents.^18^ Data collation and analysis was performed using Microsoft Excel and mapping visualization was undertaken using QGIS.^19,20^

### Eligibility criteria

**Figure 1.**
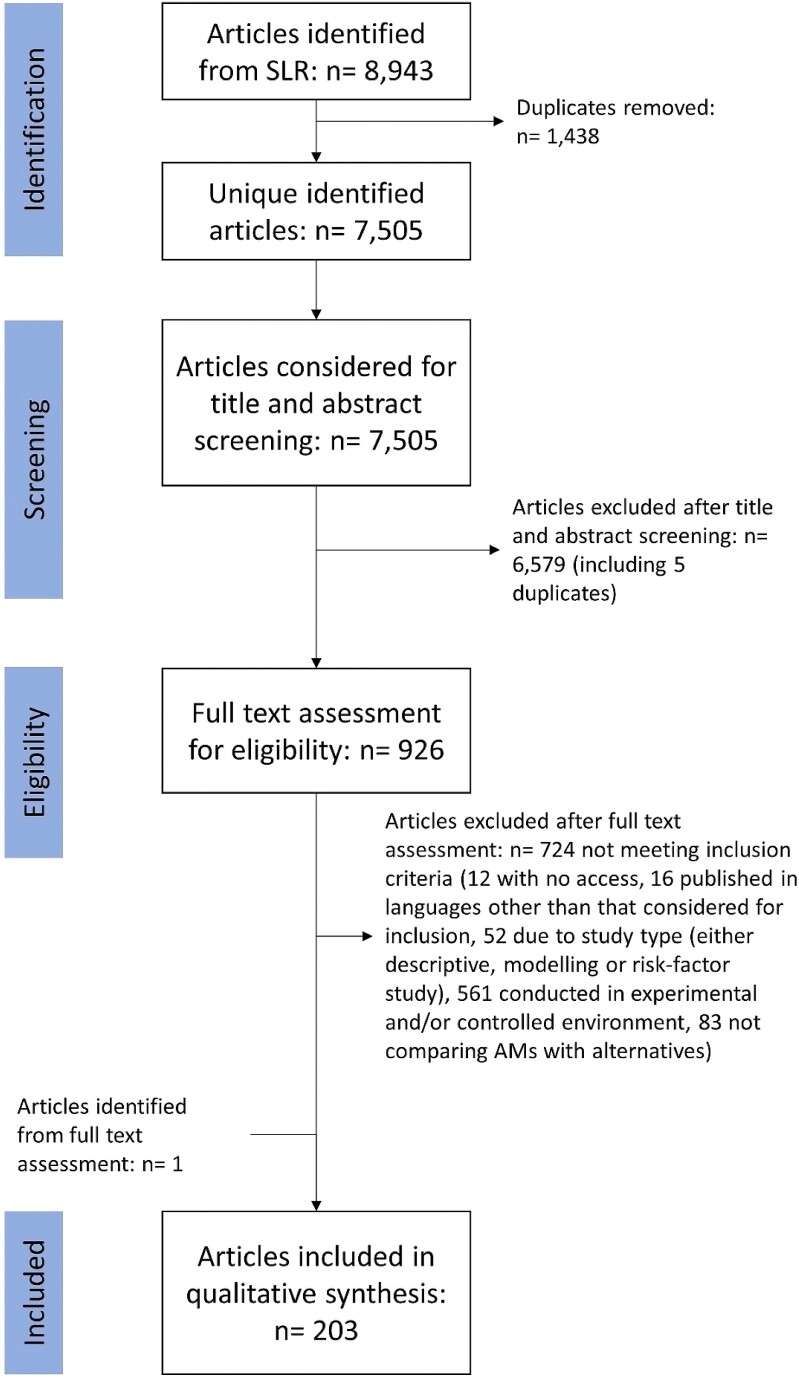
Workflow for article sifting and selection. SLR, scoping literature review.

Eligibility criteria defined in the inception table were used to sift and select articles in sysrev.com following PRISMA-ScR guidelines (see Figure 1).^15^ After deduplication, titles and abstracts were screened for eligibility, according to the criteria in Table 2.

**Table 2.** Inclusion and exclusion criteria for the identified research articles during screening stage

Criteria	Inclusion	Exclusion
Language	English, French, Italian, Portuguese or Spanish	Other than those mentioned in the Inclusion column
Document type	Peer-reviewed articles	Grey literature and other non-peer-reviewed articles
Study type	Original *in vivo* research	Reviews, *in vitro* and *in silico* research, population-based modelling research
Study population	Cattle, poultry, pigs, fish, small ruminants	Other than those mentioned in the Inclusion column
Study context	Field production context	Other than those mentioned in the Inclusion column (note: research facilities-based studies were excluded)
Study purpose	To evaluate the impact on AMU, production, animal health, economic performance and AMR of strategies to reduce AMs in livestock, including aquaculture species (note: studies in which groups with AMs were challenged with negative controls—groups not using AMs nor any other alternative or using placebos—were also considered)	Other than those mentioned in the Inclusion column

Data were extracted from articles meeting inclusion criteria. Each study was independently assessed by two reviewers. Conflicts were solved after consensus was reached between the reviewers or, when not possible, by a third reviewer. Relevant studies not captured by the search strategy but identified during full-text assessment were included in the review. Studies in which groups with antimicrobials were challenged with negative controls (groups not using antimicrobials nor any other alternative or placebo) were also considered.

### Data extraction and management

Data were extracted from screened articles by a single reviewer using a bespoke form, after consensus was reached on data parameters and approach (see Table 3). Only data available within articles were used; no attempt was made to retrieve unpublished/unclear data and article authors were not contacted. Given the heterogeneity in the way alternatives to antimicrobials or interventions to reduce AMU were described across articles, there was a need to standardize these by grouping them into categories.

**Table 3.** Parameters for data extraction

Category	Parameters
General characteristics	Author(s)
	Geography
	Language
	Title
	Year of publication
Study description	Alternative(s) and/or intervention(s)
	Antimicrobial(s)
	Number of farms
	Number of animals
	Number of study units^a^
	Outcome(s) of interest
	Study design
	Study unit(s)
	Study type
Study population	Breed
	Production purpose
	Species
Results	Effect of alternative or intervention on outcome(s) of interest

^a^This could be equal to number of farms or number of animals. However, it could be that the study unit was hoof, meaning that the number of study units would be larger than number of animals.

#### Categorization of alternatives to antimicrobials and interventions to reduce their use

*New therapy protocols* included selective dry cow treatment, reduced antimicrobial dosage, different administration routes (e.g. parenteral versus local/regional) or use of treatment decision-supporting tools. This category also included studies comparing a group treated solely with antimicrobials with groups treated with antimicrobials and supportive treatment (e.g. NSAIDs), and a study comparing a group treated solely with antimicrobials with groups treated solely with supportive treatment. Farm management decisions, such as type of production system (e.g. organic versus conventional), practising quarantine, defining animal stocking density, implementing foot-trimming and/or foot-bathing regimens, were categorized as *Farm management measures*. Interventions aimed at advising producers on managing/planning animal health and welfare and/or providing training were categorized as *Animal health advisory/training*. Several studies challenged groups treated with antimicrobials with a non-treated group. For these studies, the *No use of antimicrobials (AMs)* category was created as an alternative to antimicrobials. All other interventions were categorized as *Other*. Table 4 presents the different alternatives to antimicrobials and interventions to foster their prudent use, and how they were categorized. For ease of reading, categories are presented in alphabetical order, apart from the *Other* category, which is listed as the last row of the table.

**Table 4.** Categorization of alternatives to antimicrobials and interventions to reduce AMU identified within the review

Category	Alternative/intervention
Animal health Advisory/training	Coaching; farm field schools; herd-specific interventions defined with health expert(s); training; health expert guidance; education manual/handbook; media and newsletters; broadcast information; posters; increasing awareness
Bioactive protein and peptides	Bovine lactoferrin; lysozyme; ovotransferrin; porcine β-defensin 2; nisin
Farm management	Non-regulated antibiotic(s) withdrawal or reduction (farmer decides to change production practices); disinfectant for topical treatment; foot-bathing; foot-trimming; improved sanitation; organic farming; ‘outdoor veal calf’ concept; prophylactic measures; quarantine; stocking density
Feed/water management	Colostrum supplementation; rice and other cereal grains; medium-chain fatty acids; non-medicated milk replacer; water electrolyzed oxidation
New therapy protocol	Administration route/place; alternative topical treatment; AMU policy; best practices guidelines; flunixin meglumine; hormones and NSAIDs; delayed treatment and monitoring; delayed treatment based on on-farm diagnostic test; drug dosage reduction; laser irradiation; selective treatment based on an algorithm; selective treatment based on on-farm diagnostic test; targeted selective treatment based on scoring system; targeted treatment based on clinical symptoms; teat dipping; teat sealant
No use of AMs	Group of animals where placebo was administered or where no antimicrobials were administered, nor any other alternative to antimicrobials (these were compared against groups of animals that received antimicrobials).
Plant-based (phytogenic)	Essential oils; plant extract; plant-based teat sealant
Prebiotic	Chitosan; mannan oligosaccharide (MOS); short-chain fatty acids
Probiotics	*Lactobacillus*; *Enterococcus*; *Bifidobacterium*
Regulation	Ban of in-feed antibiotics; ban of AMU for growth promotion; ban of blanket application of antimicrobial dry cow treatment; public–private agreement; regulation
Vaccines	Vaccination against different type of pathogens
Other	Acoustic pulse therapy; bee venom; dextrose; homeopathy; intra-uterine organic product; laser irradiation; manual removal of fetal membranes; natural anti-inflammatory; nitric oxide; ozone; photodynamic therapy; rubber mats and zinc ointment; veterinary product not classified as antimicrobial

#### Antibiotic renaming and classification according to drug class and level of importance

Antibiotic names were standardized and grouped according to meaningful categories, providing insights into the different types of antibiotics used. Each standardized group was then grouped into classes according to WOAH.^21^ If an antibiotic could not be found in the WOAH list, a web search for synonyms was conducted. For example, bambermycin is also referred to as flavomycin, moenomycin and flavophospholipol.^22^ Sulfamethazine was grouped with sulfadimidine, as these are synonyms.^23^ If this was not possible, a new antibiotic class was created. Once standardized, the antibiotic groups were classified according to their level of importance from a ‘Human, Animal and One Health’ perspective, as per WHO, WOAH and Venkateswaran (unpublished work) descriptions, respectively (see Table S5 in Section 4 of the Supplementary data).^21,24,25^

#### Categorization of study outcomes into groups describing alternatives to antimicrobials or interventions to reduce AMU

There was substantial diversity across the cohort of studies in outcomes describing production and economic performance, and clinical and epidemiological impacts. As a result, these eco-epidemiological (eco-epi) outcomes were grouped into seven categories—*production*, *product quality*, *AMU*, *economic performance*, *AMR*, *clinical*, *epidemiology* (see Table 5)—which were used to assess marginal benefits and costs of adopting changes in production practices.^26^ For example, the *production* eco-epi outcome group included indicators such as milk yield, feed conversion ratio (FCR) and pregnancy rate (see Table 5). Table S3 describes the results of the qualitative assessments for individual studies.

**Table 5.** Grouping eco-epi outcomes identified from the research articles cohort included in data extraction and used to assess the impact of alternatives and interventions to reduce AMU

Eco-epi outcome group	Description of related outcomes
AMR	Level of resistance to antimicrobials, prevalence of resistant pathogens
AMU	Use of antimicrobials, treatment frequency/rate etc.
Clinical	Symptoms, lesion occurrence and treatment outcomes (e.g. cure rate, treatment failure), pathogen shedding and/or count
Economic performance	Economic performance of the production units and/or costs of adopted measures (e.g. gross margin, cost-effectiveness/benefit, costing of action)
Epidemiology	Prevalence/incidence of health/disease events and mortality, pathogen prevalence
Product quality	Quality of products from animal sources (e.g. carcass quality, SCC)
Production	Animal’s productivity and/or lifespan (e.g. milk yield, FCR, offtake rate, pregnancy rate)

SCC, somatic cell count.

#### Impact of alternatives to antimicrobials or interventions to reduce usage

**Figure 2.**
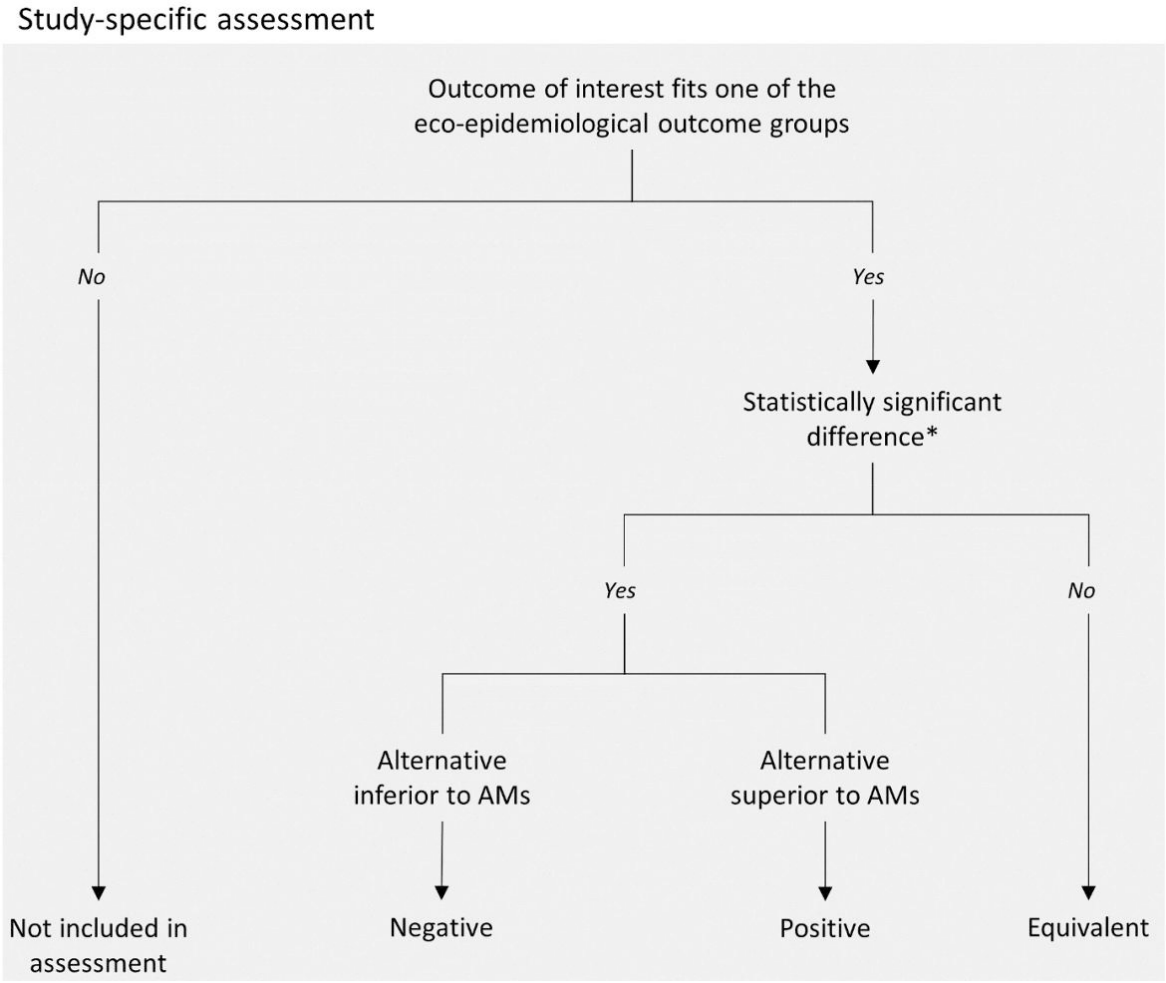
Flowchart describing decision-making on the direction of effect of the alternatives or interventions on study-specific eco-epi outcomes (*P < 0.05).

The impact of alternatives to antimicrobials and interventions to reduce their use was assessed qualitatively. Impacts relative to study-specific eco-epi outcomes (see Table 5) were classified as negative, positive or equivalent, according to the direction of the effect comparing group(s) with alternative/intervention with groups without alternative/intervention, and dependent on the statistical significance of the findings. The classification would also depend on the type of indicator and what alternatives/interventions are expected to result in. For example, if a study indicated a positive statistically significant (*P* < 0.05) difference in the FCR between the intervention group (adopting the alternative to antimicrobials or implementing the intervention to reduce its usage) compared with the group using antimicrobials, the impact for that particular eco-epi outcome would be classified as positive. On the other hand, if the study indicated a negative statistically significant difference, the impact would be classified as negative. When considering AMU as the indicator, if the study indicated a negative statistically significant difference when comparing the intervention group with the group without intervention, the impact would be classified as positive. If it was the other way around it would be considered negative. If the *P* value for the observed difference was equal to or above 0.05, the impact would be considered equivalent, even if the study reported differences between groups for a particular eco-epi outcome (Figure 2). By equivalent we mean that replacing antimicrobials with alternatives or reducing their usage through interventions resulted in no impact on the outcome, offering an adequate transition for producers to consider.

**Figure 3.**
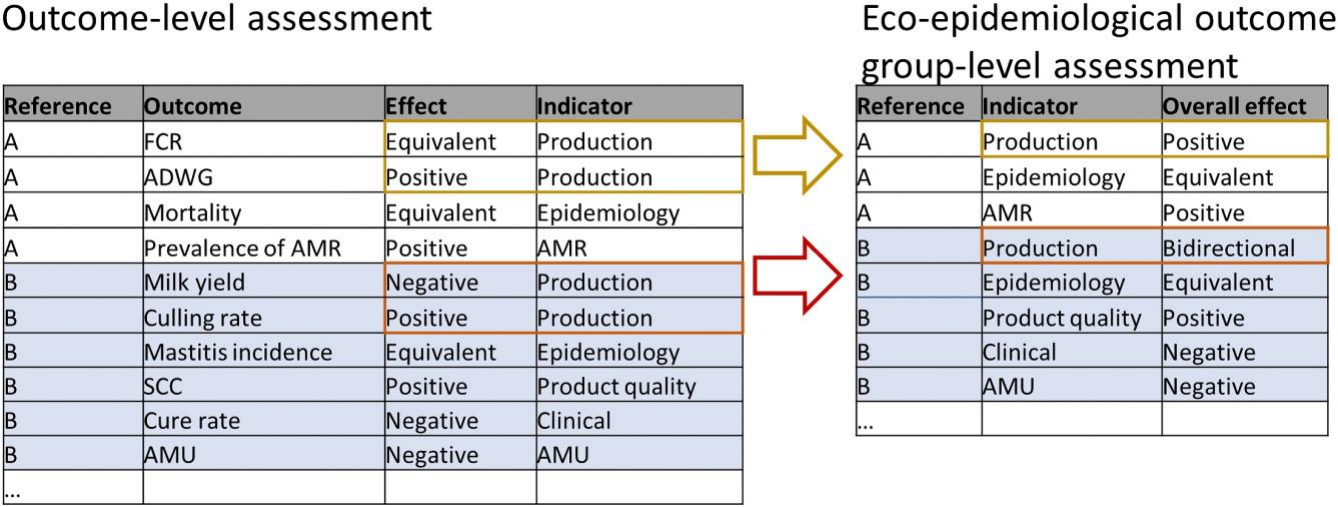
Example of the impact of qualitative assessment on eco-epi outcome groups based on the qualitative assessment of outcomes. To summarize, the alternative or intervention studied in reference A had a positive impact on the eco-epi outcome group (given that the effect on one of the outcomes within this group was equivalent and the other was positive), whereas in reference B the impact of the alternative or intervention on eco-epi outcome group was classified as bidirectional given that animals in the intervention group had a poorer milk yield compared with group(s) using antimicrobials, but also a lower culling rate. This figure appears in colour in the online version of *JAC* and in black and white in the print version of *JAC*.

Often studies would report more than one outcome per eco-epi outcome group. For example, work investigating the impact of alternatives or interventions in pig production reported average daily weight gain (ADWG), average daily feed intake (ADFI) and FCR. In such cases, the eco-epi outcome group would be classified as negative, if the effect for at least one study outcome was negative and none was positive, or as positive if the contrary was observed. If there was evidence of different outcomes within an eco-epi outcome group causing both positive and negative effects, the impact was classified as bidirectional (Figure 3). Using this method, the number of times eco-epi outcome groups are assessed qualitatively is larger than the total number of research articles, as studies can report multiple outcomes from different eco-epi outcome groups. The denominator for the qualitative assessment of indicators is the total count of assessments.

## Results

In total, 8943 research articles were identified by running searches in the three bibliometric databases. The majority of research articles were identified using MEDLINE (59.1%), with Scopus capturing 29.5% of articles and CAB Abstracts contributing 11.4%. After deduplication, a total of 7505 articles were considered for screening of titles and abstracts. Of these, 926 were eligible for full-text screening, after which 203 were considered for the qualitative evidence synthesis (including one article not captured by the search strategy) and included in full data extraction (Figure 1). A list of articles and description of their general characteristics is provided in Table S2.

### Summary statistics

**Figure 4.**
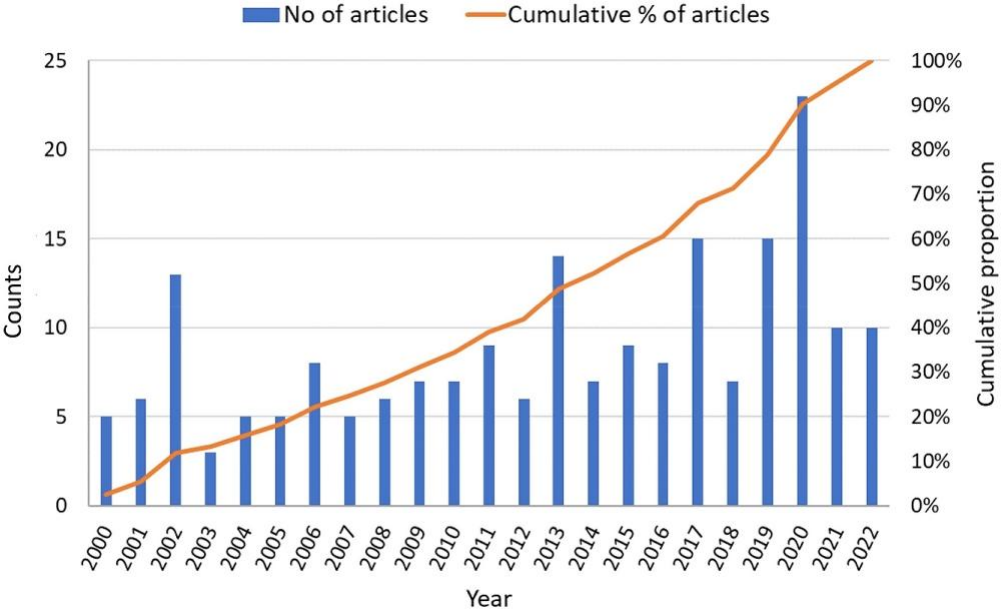
Number of research articles, by publication year, included for data extraction in the review of alternatives to antimicrobials or interventions to reduce their use in livestock and aquaculture. This figure appears in colour in the online version of *JAC* and in black and white in the print version of *JAC*.

**Figure 5.**
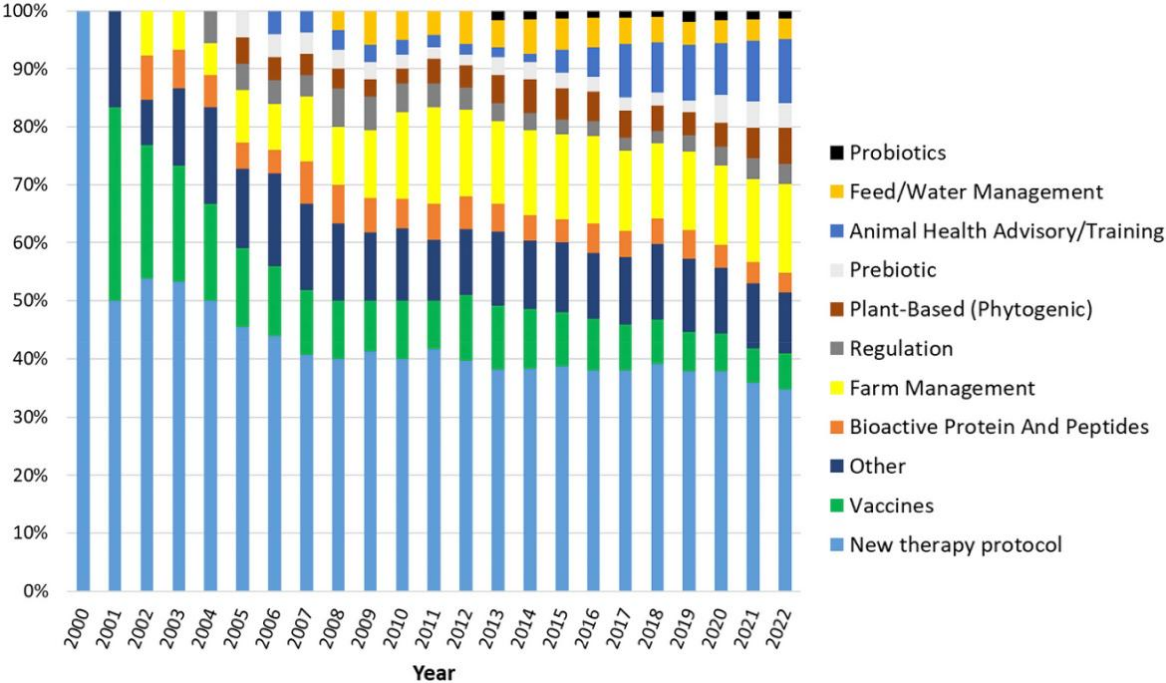
Cumulative proportion of research articles by publication year and categories describing groups of alternatives to antimicrobials or interventions to reduce AMU. Analysis excludes the category *No use of AMs.* This figure appears in colour in the online version of *JAC* and in black and white in the print version of *JAC*.

**Figure 6.**
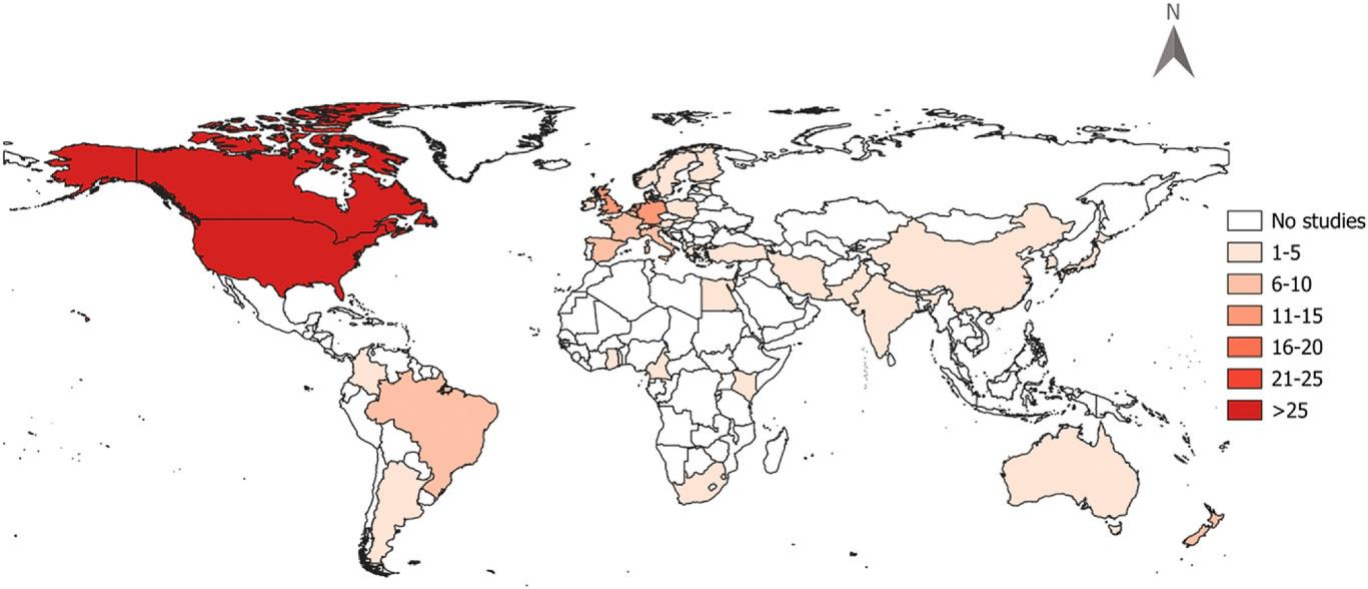
Geographical distribution of assessments of alternatives to antimicrobials or interventions to reduce AMU in livestock and aquaculture described within research articles. This figure appears in colour in the online version of *JAC* and in black and white in the print version of *JAC*.

The earliest article included in data extraction was published in the year 2000, and more than half of articles were published from 2013 onwards (Figure 4). When excluding articles with *No use of AMs*, up to the year 2004, with few exceptions the use of vaccines and new therapy protocols dominated research, representing two-thirds of the cumulative proportion of publications. The frequency of studies measuring the impact of vaccines and new therapy protocols reduced over time; this contrasted with other alternatives and interventions, particularly interventions based on animal health advice and training, improved farm management practices and plant-based products (Figure 5). Most studies were intervention-based (90.1%) and the majority (78.3%) were focused on AMU in Europe or North America (Table 6), with an uneven distribution of assessments across countries (Figure 6). One hundred and thirteen articles (55.7% of studies) included dairy cattle as the study population, followed by pigs (19.2%) and beef cattle (9.4%). The least represented species were goats, turkeys and tilapia, each with one article (0.5%). Antibiotics were the most commonly examined type of antimicrobial (92.1% of research articles), followed by anthelmintics (3.9%; Table 6).

**Table 6.** Characteristics of research articles in the review of alternatives to antimicrobials and interventions to reduce AMU

Category	Number and proportion (%) of research articles
Study type	
Analytical intervention-based	194 (90.1)
Analytical observational	9 (9.9)
Region according to WOAH	
Africa	7 (3.4)
Americas	78 (38.4)
Asia, Pacific and Oceania	21 (10.3)
Europe	91 (44.8)
Middle East	4 (2.0)
Multiple	1 (0.5)
Unknown	1 (0.5)
Region according to WHO—UN SDG1	
Australia and New Zealand	11 (5.4)
Central and Southern Asia	3 (1.5)
Eastern and South-Eastern Asia	8 (3.9)
Europe and North America	159 (78.3)
Latin America and the Caribbean	10 (4.9)
Multiple	1 (0.5)
Northern Africa and Western Asia	6 (3.0)
Sub-Saharan Africa	4 (2.0)
Unknown	1 (0.5)
Region according to WHO—UN SDG2	
Australia and New Zealand	11 (5.4)
Eastern Asia	8 (3.9)
Europe	91 (44.8)
Multiple	1 (0.5)
North America	68 (33.5)
Northern Africa	3 (1.5)
South America	10 (4.9)
Southern Asia	3 (1.5)
Sub-Saharan Africa	4 (2.0)
Unknown	1 (0.5)
Western Asia	3 (1.5)
Animal species/purpose	
Beef cattle	19 (9.4)
Broiler	10 (4.9)
Dairy cattle	113 (55.7)
Goat	1 (0.5)
Layer	3 (1.5)
Multiple	4 (2.0)
Sheep	12 (5.9)
Pigs	39 (19.2)
Tilapia	1 (0.5)
Turkey	1 (0.5)
Antimicrobials studied	
Antibiotics	187 (92.1)
Antifungals	1 (0.5)
Anthelmintics	8 (3.9)
No antimicrobials used	6 (3.0)
Other	1 (0.5)

SDG, sustainable development goal.

**Figure 7.**
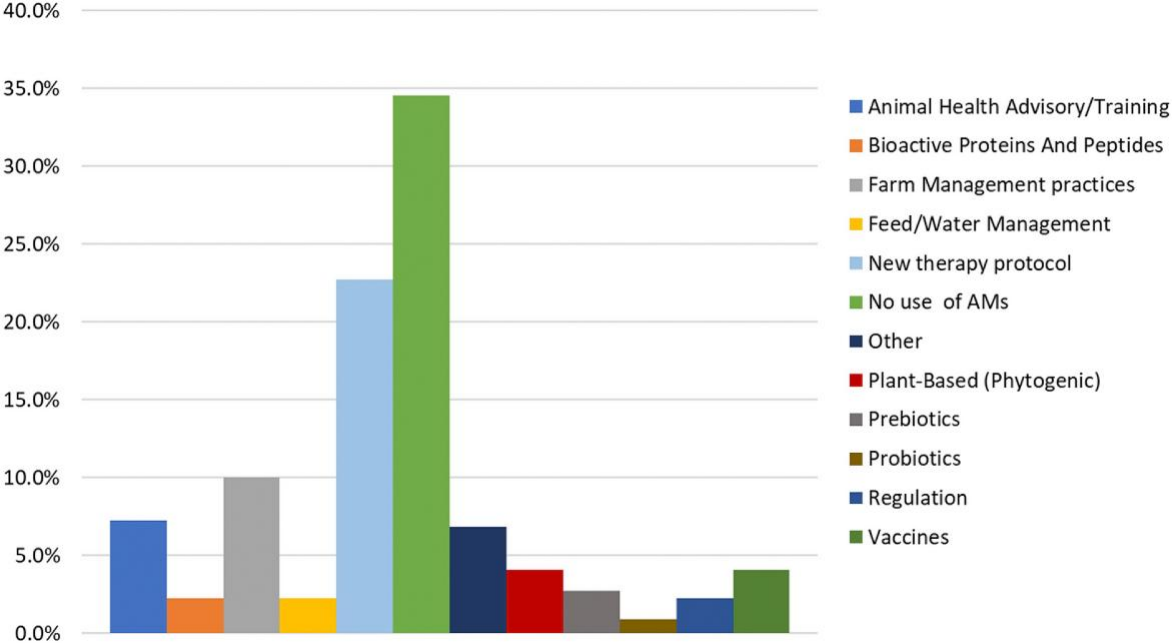
Relative distribution of alternatives to antimicrobials or interventions to reduce AMU described in research articles. Note: some articles included more than one alternative or intervention, thus the total count of assessments is larger than the total number of articles. This figure appears in colour in the online version of *JAC* and in black and white in the print version of *JAC*.

Of the alternatives or interventions to AMU that were examined, one-third compared groups not using antimicrobials (groups not using antimicrobials or using placebo) with groups still using them (34.5%), and a quarter explored the impact of new therapy protocols (22.7%). Probiotics were the least represented and were only examined twice (0.9%; Figure 7).

Alternatives or interventions were most often investigated in dairy cattle (with alternatives or interventions from 12 different categories examined), followed by pigs (10) and broiler chickens (7). Goats, tilapia and turkeys each had a single alternative or intervention examined. Use of probiotics was only examined in dairy cattle and boiler chickens. Four out of the five studies examining *Regulation* interventions were in pigs, and the other study was in dairy cattle (Table 7).

**Table 7.** Distribution of alternatives to antimicrobials or interventions to reduce AMU from the research articles according to animal species and production purpose, where relevant

Alternative/intervention	Frequency	Species/production system (row %)									
		Beef cattle	Broiler	Dairy cattle	Goat	Layer	Multiple	Sheep	Pigs	Tilapia	Turkey
Animal health advisory/training	16	6.3	12.5	31.3	—	6.3	—	—	43.8	—	—
Bioactive protein and peptides	5	—	—	60.0	—	—	—	—	20.0	—	20.0
Farm management	22	18.2	4.5	54.5	—	4.5	—	9.1	4.5	4.5	—
Feed/water management	5	—	20.0	20.0	—	—	20.0	—	40.0	—	—
New therapy protocol	50	4.0	—	88.0	—	—	—	8.0	—	—	—
No use of AMs	76	14.5	3.9	50.0	1.3	—	1.3	7.9	21.1	—	—
Other	15	6.7	—	73.3	—	—	—	—	20.0	—	—
Plant-based (phytogenic)	9	—	—	44.4	—	11.1	22.2	11.1	11.1	—	—
Prebiotic	6	—	33.3	33.3	—	—	—	—	33.3	—	—
Probiotics	2	—	50.0	50.0	—	—	—	—	—	—	—
Regulation	5	—	—	20.0	—	—	—	—	80.0	—	—
Vaccines	9	11.1	11.1	11.1	—	—	—	22.2	44.4	—	—

The antibiotic classes most often examined were tetracyclines, natural penicillins, macrolides (14-, 15- and 16-membered ring) and anti-staphylococcal penicillins, representing 11.7%, 11.2%, 9.1% and 7.6% of the total number of records for different antibiotic classes (*n* = 197), respectively. The least often examined were bicyclomycin, diaminopyrimidines, orthosomycins and quinoxalines, each accounting for 0.5% (Figure S1). When considering their importance for public health according to WHO, 93.4% of the antibiotics were either highly important or important; this proportion was 95.9% using WOAH’s classification.^24^ Based on Venkateswaran,^25^ 52.8% of antibiotics were relevant from a One Health perspective (Figure S2).

### Alternatives to antimicrobials or interventions to reduce AMU and their impacts

Different eco-epi outcome groups were qualitatively assessed 485 times in total. When all alternatives or interventions were considered, 69.7% had either an equivalent or a positive impact on the eco-epi outcome groups considered. This proportion increased to 84.1% when the studies comparing groups not using antimicrobials with groups still using them were excluded (Table S5).

**Figure 8.**
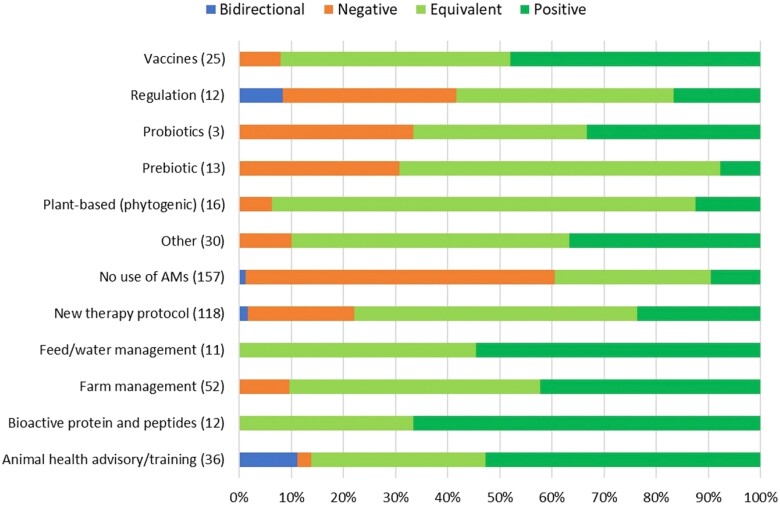
Relative distribution and direction of impacts of alternative or intervention categories upon eco-epi outcome groups related to livestock and aquaculture production (number of assessments within parentheses). This figure appears in colour in the online version of *JAC* and in black and white in the print version of *JAC*.

Examining the impact across specific alternatives or interventions categories, the *No use of AMs* category had the highest proportion of negative impacts (59.2%), followed by *Probiotics*, *Regulation* and *Prebiotics* (33.3%, 33.3% and 30.8%, respectively). Of the alternative or intervention categories linked to positive impacts on eco-epi outcome groups, *Bioactive protein and peptides*, *Animal health advisory/training* and *Feed/water management* had the highest proportions (66.7%, 54.5% and 52.8%, respectively). *Plant-based (phytogenic)*, *Prebiotics* and *New therapy protocol* were most strongly linked to equivalent impacts amongst alternative or intervention categories (81.3%, 61.5% and 54.2%, respectively). Of all the alternative or intervention categories, *Animal health advisory/training* was most strongly linked to bidirectional impacts (11.1%) followed by *Regulation* (8.3%). *Bioactive protein and peptides* and *Feed/water management* categories had no reports of negative or bidirectional impacts (Figure 8 and Table S5). Antimicrobial and intervention impacts upon eco-epi outcome groups were examined in more detail for cattle, pigs and broiler chickens (with a summary of results for all species provided in Table S6). Additionally, the impacts of alternatives to antimicrobials and interventions to reduce their use according to eco-epi outcome group were also investigated and are presented in Table S7.

#### Beef cattle

**Figure 9.**
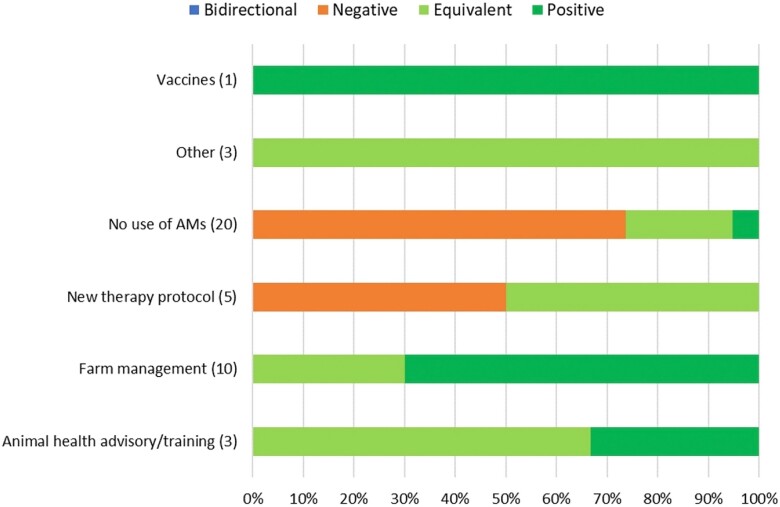
Relative distribution and direction of impacts of alternative or intervention categories upon eco-epi outcome groups related to beef production (number of assessments within parentheses). This figure appears in colour in the online version of *JAC* and in black and white in the print version of *JAC*.

The impacts on eco-epi outcome groups of 42 separate evaluations within six alternative or intervention categories were examined for beef cattle. Apart from *New therapy protocol* and *No use of AMs*, all alternative or intervention categories had either equivalent or positive impacts on eco-epi outcome groups. Shifting therapeutical practices from using antimicrobials to not using them nor any other alternative (*No use of AMs*) resulted most often (70% of the time) in negative impacts. *New therapy protocol* had negative impacts on eco-epi outcome groups 40% of the time they were assessed (Figure 9 and Table S6).

#### Broilers

**Figure 10.**
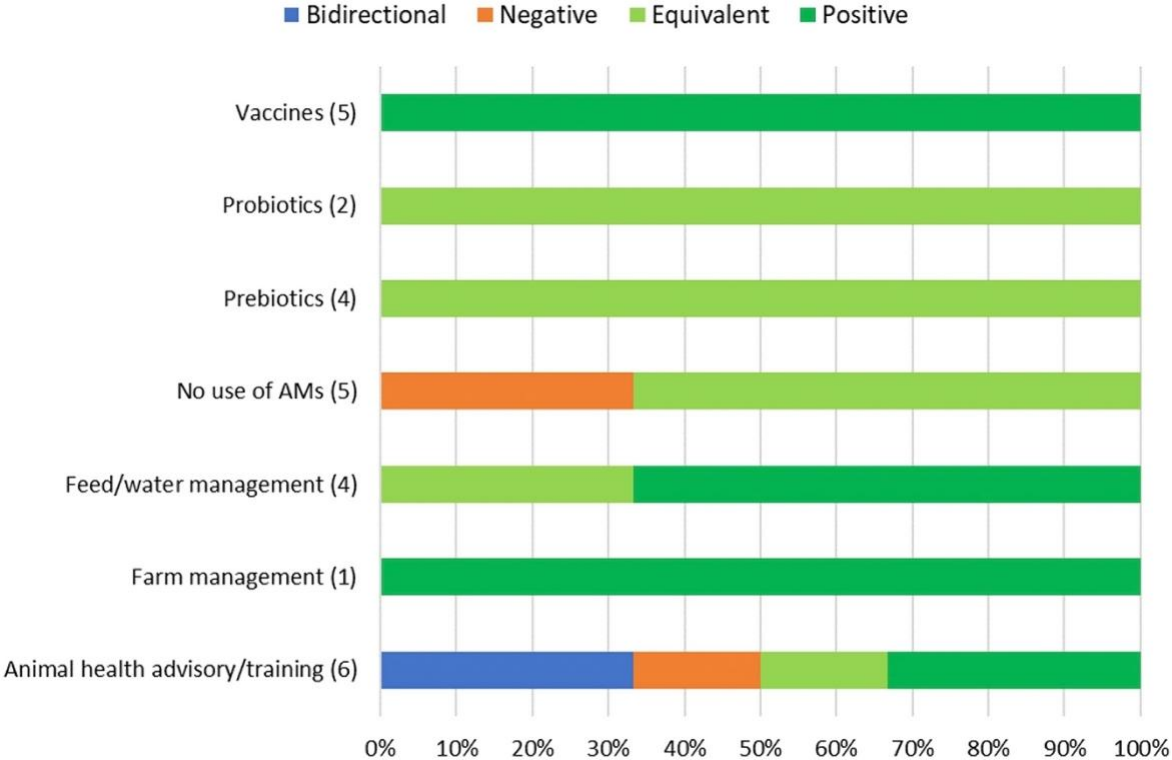
Relative distribution and direction of impacts of alternative or intervention categories upon eco-epi outcome groups related to broiler production (number of assessments within parentheses). This figure appears in colour in the online version of *JAC* and in black and white in the print version of *JAC*.

The impacts on eco-epi outcome groups of 27 separate evaluations within seven alternative or intervention categories were examined for broiler chickens. With the exception of *No use of AMs* and *Animal health advisory/training*, all alternative or intervention categories had equivalent or positive impacts on broiler eco-epi outcome groups. There were negative impacts of *No use of AMs* upon eco-epi outcome groups one-fifth of the time. This was also observed for positive effects, whereas the majority of the time (60%) *No use of AMs* resulted in an equivalent effect. *Animal health advisory/training* resulted in contrasting impacts across articles; bidirectional (within the same study) or positive impacts were identified one-third of the time, respectively, and the residual was split between equivalent or negative impacts (Figure 10 and Table S6).

#### Dairy cattle

**Figure 11.**
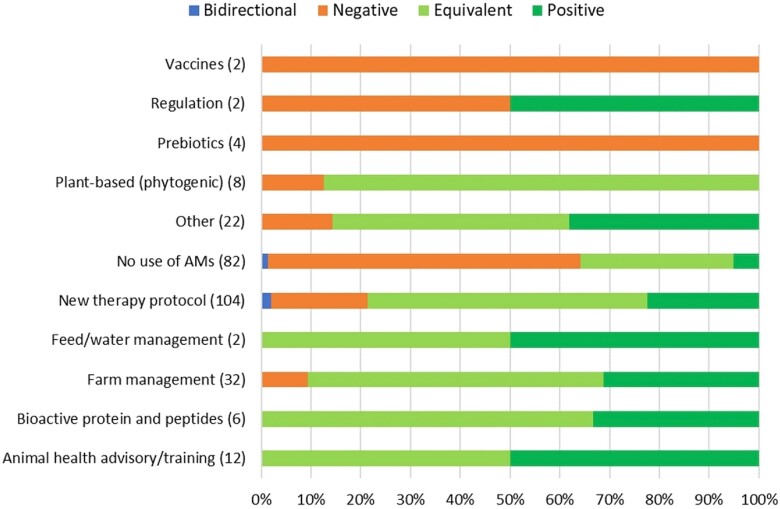
Relative distribution and direction of impacts of alternative or intervention categories upon eco-epi outcome groups related to dairy cattle production (number of assessments within parentheses). This figure appears in colour in the online version of *JAC* and in black and white in the print version of *JAC*.

The impacts on eco-epi outcome groups of 277 separate evaluations within 12 alternative or intervention categories were examined for dairy cattle. *Probiotics* and *Prebiotics* always resulted in negative impacts on eco-epi outcome groups. In contrast, *Bioactive proteins and peptides*, *Feed/water management* and *Animal health advisory/training* had either equivalent or consistently positive impacts. Four-fifths of the time *New therapy protocol* category was assessed resulted in either equivalent or positive impacts on eco-epi outcome groups, with similar patterns for *Plant-based (phytogenic)* and *Other* categories. *Regulation* had inconsistent results, with evaluations resulting in both positive and negative impacts (Figure 11 and Table S6).

#### Pigs

**Figure 12.**
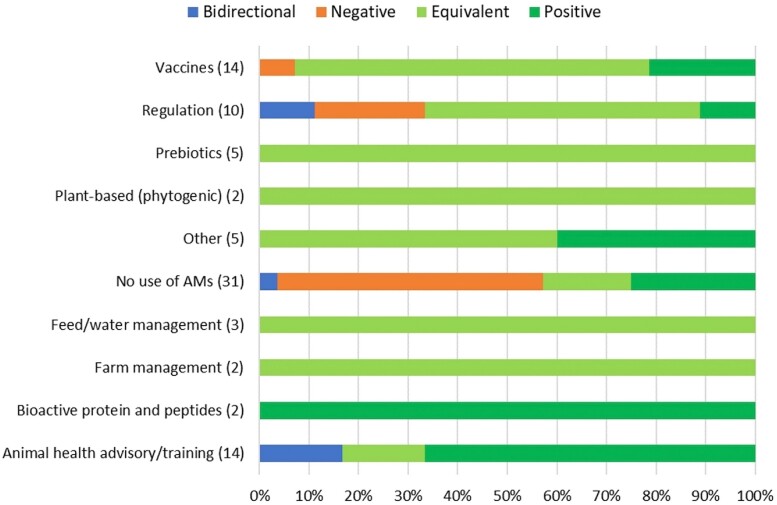
Relative distribution and direction of impacts of alternative or intervention categories upon eco-epi outcome groups related to pig production (number of assessments within parentheses). This figure appears in colour in the online version of *JAC* and in black and white in the print version of *JAC*.

The impacts on eco-epi outcome groups of 88 separate evaluations within 10 alternative or intervention categories were examined for pigs. *Prebiotics*, *Plant-based (phytogenic)*, *Bioactive proteins and peptides*, *Feed/water management*, *Farm management* and measures classified as *Other* had equivalent or positive impacts on eco-epi outcome groups. *Vaccines* and *Animal health advisory/training* led to largely positive or equivalent impacts (92.9% and 85.7%, respectively). *Regulation* and alternatives classified as *No use of AMs* had the lowest proportion of positive or equivalent impacts. *No use of AMs* had negative impacts on eco-epi outcome groups more than half the time (54.8%), and *Regulation* had negative impacts 30.0% of the time (Figure 12 and Table S6).

## Discussion

This research assessed the impact of alternatives to antimicrobials and interventions aimed at reducing their usage in livestock production and aquaculture. In general, most studies reported positive or equivalent (neutral impact meaning that performance for the corresponding eco-epi outcome group is not expected to change if strategies to replace antimicrobials or to reduce their use are adopted) impacts on eco-epi outcome groups in livestock and aquaculture. This includes the use of vaccines, feed and water management, bioactive molecules and peptides, plant-based (phytogenic) products, and changing farm management decisions. Conversely, caution is needed when applying new therapy protocols, using prebiotics or probiotics, relying on regulation or simply stopping using antimicrobials altogether without replacing them with an alternative health management tool as, according to the study results, the impact is likely to be negative. When examining results for species separately (recognizing variability in the number of assessments for each species), there were differences in the impacts of alternative treatments or interventions used. Farm management, and feed and water management practices provided (approximately) consistent results, with all assessments of dairy and beef cattle, broilers and pigs resulting in positive or equivalent impacts, other than for three farm management assessments. Overall, these results suggest that reductions in AMU are possible in food-producing species; however, antibiotic replacement using alternatives or interventions need careful species-specific management. This is particularly relevant when using regulation-based interventions. Enforcing changes to production practices might lead to severe impacts at farm level, affecting production and the livelihoods of producers, with ripple effects across the value chain all the way to consumers. A thorough understanding of the cost–benefit of alternatives to antimicrobials and interventions to reduce its use will help regulators make evidence-based decisions, indicating when producers are expected to adjust their production practices, and when they need to be supported to make those changes. Also, evidence-based decision-making requires data, which this research suggests are lacking. More research is required to investigate the impacts of alternatives to antimicrobials and interventions to reduce their usage in livestock and aquaculture production.

The above considerations are important when highlighting the need for change, particularly in the context of potential external threats such as the COVID-19 pandemic and, more recently, war in Europe, that can result in inflation and disruptions in the supply chain and prices. Animal food producers are likely to be unwilling or unable to change their farming practices if the required changes are not economically viable or are likely to come at a high cost, and these external threats are likely to increase the risk of inaction by these actors. This is particularly important for AMU, considering its affordability, accessibility and effectiveness in managing animal health. For example, in broiler production, research has highlighted that medicines overall are a low percentage of total production costs. Therefore, low prices of antimicrobials may drive their routine use by broiler producers.^27–29^ Reducing or banning AMU in livestock and aquaculture production systems without an effective alternative can compromise the financial success and therefore livelihoods of producers. This is exacerbated by uneven distribution of profits across the food supply chain, with producers perceived as the least rewarded and taking most often the role of price-takers.^30–34^ If change is to be promoted, producers must have tools to replace antimicrobials in a cost-effective manner that balances farm-level impacts and costs with externalities such as societal benefits. The responsibility for AMU and AMR lies with all food-chain actors and also with consumers; holistic approaches are needed.

More than 50% of studies used critically important antimicrobials according to WHO; this rose to 70% for WOAH. This research interest could be due to the need to find alternatives to this particular category of antimicrobials; however, it could also reflect particular classes of antimicrobial still being widely used in livestock production and aquaculture. In addition, there is heterogeneity in the potential selective pressure for resistance, and its persistence in the environment; this is particularly important when considering usage and likely impacts on emerging resistance from a One Health perspective because, for example, prescribing stakeholders focused on human health are unlikely to consider the impacts of their prescriptions on selection pressures in the environmental and veterinary domains. This is demonstrated by metabolites being present in urine from cattle after parenteral administration of ceftiofur; they have been shown to amplify resistance in *Escherichia coli* populations, potentially impacting persistence in the environment. Florfenicol and β-lactam antimicrobial residues have also been shown to negatively impact the soil microbiome, whereas ciprofloxacin, neomycin and tetracyclines were neutralized.^35–37^ Development of AMR increases with use, jeopardizing the effectiveness of these tools in managing both human and animal health. Further efforts are needed to help producers reduce the level of AMU, to foster more sustainable food supply chains and safeguard public health. In particular, the development and/or strengthening of systems designed to collect data at the most granular levels possible to monitor AMU are vital to help us understand the extent of the problem and the longitudinal trends.

Most studies were conducted in high-income regions, with Europe and North America accounting for nearly 80% of total publications (Table 6). According to WOAH, the region with the highest estimated usage of antimicrobials per kg of livestock biomass in 2018 was Asia, Far East and Oceania; this is 62.3% higher than the second highest region—Europe. AMU for growth promotion was also reportedly uneven across different regions, with Europe having the lowest (proportion of member state) (3.7%), followed by Africa (20.4%), Asia (31.3%), Far East and Oceania and Americas (51.5%).^38^ This is concerning, indicating uneven research efforts and presumably funding in different regions of the world. It’s also unsurprising, given the heterogeneity in regulation on AMU in livestock and aquaculture.

Our results suggest that dairy cattle are most often studied, accounting for more than 50% of articles. This could be due to many different factors, such as higher interest for particular study populations, their economic relevance or species-specific diversities in alternatives to antimicrobials. It could also reflect the structure of livestock and aquaculture sectors, with each sector having particular levels of integration and cooperativism. The aquaculture, poultry and pig sectors are highly integrated businesses for example. Having tighter control of the supply chain would, in principle, allow an operator to be less dependent on cooperation, controlling the resources needed for improving the operations and exploring alternatives to antimicrobials. So, it could be that these sectors of livestock industry are making use of these tools but not sharing conclusions from their field trials for the sake of market competition. In the dairy sector there is uneven bargaining power, which causes competition across the milk supply chain, meaning that ‘average’ dairy producers can struggle to make profits. It is therefore more important for them to try to reduce animal health management costs (perhaps establishing partnerships with researchers) to increase profitability. Additionally, research institutes commonly have close ties with dairy farms (with some having their own); the majority of articles rejected during full-text assessment were due to their setting context being experimental and controlled dairy units. This was also true for aquaculture settings, highlighting the need for stronger collaborations on AMU and emerging resistance between the private and research sectors.

We made a decision to include studies for which groups not using antimicrobials or using placebo were compared with groups using antimicrobials. Whilst this can be challenged as it does not provide livestock and aquaculture producers with replacement animal health management tools, it does help to capture the impact of discontinuing AMU in livestock and aquaculture production. In addition, as nearly a third of alternatives examined fall into this category, this could alter the perception of alternatives to antimicrobials. If the results of studies comparing antimicrobial-administered groups against groups without antimicrobials or with placebo were classified as negative, it would indicate that the group receiving antimicrobials outperformed the group not receiving them, but in the absence of other replacement tools. This is perceivable when looking at the figure from Table S7, with the impact of not using antimicrobials being largely negative on outcomes related to economic performance, production, product quality, and epidemiological and clinical aspects.

Our work has identified a high number of alternatives or interventions to AMU, aggregated them into meaningful groups, and qualitatively assessed their impacts. A qualitative meta-synthesis was undertaken because the heterogeneities in research methods, outcomes of interest, and antimicrobial alternatives and interventions that were examined meant that a quantitative meta-analysis could not be undertaken.^39^ Within our work we took a structured scoping review approach; we broadened search criteria to capture and provide a reasonable number of research articles for review. The objective classification system that we have developed (based on interpretation of the direction of impact and statistical significance of study results) should be interpreted with caution because it describes generalized results; those using the system need to be cognisant of specific outcome details within studies, and the size of alternative and intervention impacts. Additionally, the summarized results describe different study designs and sites, and a range of sample sizes were examined within studies. The issue of sample size is particularly relevant when the effects of alternatives or interventions were classified as equivalent, given the potential underlying poor statistical power limiting the detection of a statistically significant effect. It could be that an existing effect of the alternative or intervention is in fact unknown, and a larger sample size would indicate a significant difference, either positive or negative, from a statistical standpoint. Comparisons of impacts were thus made regardless of heterogeneities in these design variables, potentially biasing results interpretation. Non-randomized field trials would be unlikely to be representative, potentially not being generalizable beyond the study population. Well-designed studies investigating interventions to reduce AMU in randomly obtained populations would provide ideal external validity in results and a more accurate picture of expected impacts. Recently, research has examined specific therapies as alternatives to AMU.^40,41^ These studies were not well captured in our work, potentially because we focused on the field production context. This could well justify the small number of studies identified looking at probiotics, prebiotics and bioactive protein and peptides. Regarding the latter category, field trials of such technologies, like bacteriophages and peptides, may also be inhibited by high production costs and regulatory issues hampering commercialization.^41,42^

### Conclusions

This structured scoping literature review has identified and summarized evidence describing the utility of alternatives to antimicrobials or interventions to reduce their usage. Most studies reported positive or equivalent impacts on eco-epi outcome groups. Despite this, there was heterogeneity in the impact of alternative or intervention categories, highlighting the absence of a ubiquitous solution to tackle AMU and emerging resistance problems in livestock and aquaculture. Some solutions seem more promising than others, where impacts were consistently positive or equivalent when compared with groups using antimicrobials, for example *Bioactive protein and peptides, Feed/water management* and *Probiotics*. By identifying and listing the peer-review literature on alternatives and interventions to antimicrobials in livestock production under a field context, this work provides a useful resource for researchers, animal health workers and regulators to understand the most promising alternatives to antimicrobials and measures to reduce their usage. As new studies provide further data, this review can be updated. We have provided grounds to better understand alternatives to antimicrobials in a (field) livestock and aquaculture production context; this will inform cost-effectiveness model development for AMU alternatives and interventions.

## Supplementary Material

dkad350_Supplementary_Data

## Supplementary data

Figures S1 and S2, Tables S1 to S7 and Supplementary material are available as Supplementary data at *JAC* Online.
