# Supplementary material for: Strategies to reduce antimicrobials in livestock and aquaculture, and their impact under field conditions: a structured scoping literature review
Source: J Antimicrob Chemother. 2023 Nov 10;79(1):11–26. doi: 10.1093/jac/dkad350 (PMC10761277; doi:10.1093/jac/dkad350)
Supplement: dkad350_Supplementary_Data [file dkad350_supplementary_data.docx]

# Inception table

Table S.1. GBADs literature reviewing inception table

| Section and Topic | Item  # | Elements recommended for reporting | Specific review details |
| --- | --- | --- | --- |
| TITLE | 1 | - Identify the report as a systematic review in the title. - Report an informative title that succinctly provides key information about the main objective or question the review addresses including stating items such as:   - The outcome domain(s) (e.g. epidemiological/animal husbandry measures/economic/socio-economic/specific research or policy method) and specific measure(s) (e.g. prevalence/fertility/cost of medicines) to be reviewed (outcomes),   - The disease/condition of interest which the outcome measure is describing,   - The population of interest (participants),   - The geographical setting,   - The time-period being examined.   i.e. Identifying [outcome measure and domain, if necessary] in [disease/condition of interest] in [population/time-period/spatial area]. e.g. “**Identifying the prevalence of foot and mouth disease in cattle in Ethiopia between 2010 and 2019**”.   - *Consider providing additional information in the title, such as the method of analysis used, the designs of included studies, or an indication that the review is an update of an existing review, or a continually updated (“living”) systematic review.* | Effects of strategies to reduce antimicrobials in food-producing animal species and their impact under field conditions: a structured scoping literature review |
| ABSTRACT | 2 | • Report an abstract addressing each relevant item in the [PRISMA 2020 for Abstracts checklist](https://www.bmj.com/content/372/bmj.n160) (see box 2 within this). | This review aimed to identify the impact of alternatives to the use of antimicrobials and interventions aimed at reducing antimicrobial use (AMU) in livestock farming including aquaculture under a field production context, be it through productivity (e.g. a reduction in animal yield as a result of reducing AMU), an economic (e.g. costs of reducing stocking density in broiler production or cost-efficiency of administering non-steroid anti-inflammatory drugs in beef cattle for preventing BRD versus conventional use of antibiotics) or an epidemiological lenses (e.g. reduction in mortality and/or morbidity as a result of reduced health events due to vaccination)  Research articles were identified from four bibliometric databases: Cab Abstracts, Medline (via the Ovid interface) and Scopus. Articles published in languages other than English, French, Italian, Portuguese or Spanish were excluded. Additionally, articles not studying livestock population were removed. Review articles were identified to identify additional articles. |
| INTRODUCTION | | | |
| RATIONALE | 3 | Describe the rationale for the review in the context of existing knowledge - this should help readers understand why the review was conducted and what the review might add to existing knowledge.  **Essential elements**   - Describe the current state of knowledge and its uncertainties. - Articulate why it is important to do the review. - If other reviews addressing the same (or a largely similar) question are available, explain why the current review was considered necessary (for example, previous reviews are out of date or have discordant results; new review methods are available to address the review question; existing reviews are methodologically flawed; or the current review was commissioned to inform a guideline or policy for a particular organisation). If the review is an update or replication of a particular systematic review, indicate this and cite the previous review. | Limited information on the impact of alternatives to AMU. Mapping of available information would be useful and, if possible, meta-analysis could be conducted to provide pooled estimates of the different parameters. |
| OBJECTIVES | 4 | - Provide an explicit statement of all objective(s) or question(s) the review addresses, expressed in terms of a relevant question formulation framework. - It is useful to use a question formulation framework such as PEO (Population and their problems; Exposure; Outcomes or Themes - for different designs see [bmjgh-2018-001107supp001.pdf](file:///C:\Users\mcintyrm\Downloads\bmjgh-2018-001107supp001.pdf)) to state the comparisons that will be made. | **Objectives**:  Identify peer-reviewed research articles reporting the impact of interventions to alleviate the use of AM in livestock production and aquaculture, in a real production context, in any population in the world. Conduct either a qualitative meta-synthesis or quantitative meta-analysis. |
| METHODS | | | |
| ELIGIBILITY CRITERIA | 5 | - Specify all study characteristics used to decide whether a study is eligible for inclusion in the review. Consider the elements you wish to limit within search phrases, such as eligible study design(s) and setting(s) e.g. - How is the disease/condition of interest/research or policy method defined? If necessary, split into sub-concepts - Who should make the diagnosis? e.g. veterinarian, para-vet, livestock keeper - What are the most important characteristics that describe the study participants? - Are there any relevant demographic factors e.g. age, breed? - What is the setting? e.g. dairy system   - How data-rich is the topic? – If data poor, then may need to review over a longer time-period - How will studies involving only a subset of relevant participants be handled? - Specify eligibility criteria with regard to report characteristics, such as year of dissemination, language, and report status e.g. whether reports, such as unpublished manuscripts and conference abstracts, are eligible for inclusion. *NOTE: you need to be able to clearly understand and report if studies were ineligible because the outcomes of interest were not measured, or ineligible because the results for the outcome of interest were not reported. Reporting that studies were excluded because they had “no relevant outcome data” is ambiguous and should be avoided.* - *Consider providing rationales for any notable restrictions to study eligibility. For example, authors might explain that the review was restricted to studies published from 2000 onward because that was the year certain information was first available.* - Specify any groups to be used in the synthesis (e.g. outcome and population groups) and link these to the comparisons specified in the objectives (item #4). | **Inclusion criteria**:   - Food-producing animal species (cattle, poultry, swine, fish, small ruminants) - Peer-reviewed publication - English, French, Italian, Portuguese or Spanish - In vivo studies - Original research - Studies conducted in a ‘real’ production context, be it for commercial or subsistence purposes - Studies comparing AMs with alternatives (eg. Clinical trials, pre-post intervention studies, longitudinal observational studies, retrospective observational studies)   **Exclusion criteria**:   - Publication language other than the cited above - Species other than cited above - Lab-based/Research facilities-based studies - In vitro and/or in silico studies - Modelling approaches - Reviews - Studies not comparing AMs with alternatives |
| INFORMATION  SOURCES | 6 | - Specify the date when each source (e.g. database, register, website, organisation) is to be searched or consulted. - If bibliographic databases are to be searched, specify for each database its name (e.g. MEDLINE), the interface or platform through which the database is to be searched (e.g. Ovid, EBSCOhost), and the dates of coverage (where this information is provided). - If study registers, regulatory databases and other online repositories are to be searched, specify the name of each source and any date restrictions that will be applied. - If websites, search engines or other online sources are to be browsed or searched, specify the name and URL of each source. - If organisations or manufacturers are to be contacted to identify studies, specify the name of each source. - If individuals are to be contacted to identify studies, specify the types of individuals contacted (e.g. authors of studies included in the review or researchers with expertise in the area). - If reference lists are to be examined, specify the types of references to be examined (e.g. references cited in study reports included in the systematic review, or references cited in systematic review reports on the same or similar topic). - If cited or citing reference searches (also called backward and forward citation searching) is to be conducted, specify the bibliographic details of the reports to which citation searching is to be applied, the citation index or platform to be used (e.g. Web of Science), and the date the citation searching will be done for. - If journals or conference proceedings are to be consulted, specify of the names of each source, the dates covered and how they will be searched (e.g. handsearching or browsing online). | - PubMed, Cab Abstracts, Web of Science, Scopus |
| SEARCH STRATEGY | 7 | - Provide the keyword and phrase search terms to be used within the search. Split these into those describing the five sections discussed in #1 the study title, including:   - The outcome domain(s) and specific measure(s) to be reviewed,   - The disease/condition of interest which the outcome measure is describing,   - The population of interest (participants),   - The geographical setting,   - The time-period being examined. - Describe any limits applied to the search strategy (e.g. date or language) and justify these by linking back to the review’s eligibility criteria. - If natural language processing or text frequency analysis tools are to be used to identify or refine keywords, synonyms, or subject indexing terms to use in the search strategy, specify the tool(s) used. - If a tool is to be used to automatically translate search strings for one database to another, specify the tool to be used. - If the search strategy is to be validated - for example, by evaluating whether it could identify a set of clearly eligible studies - report the validation process used and specify how studies will be selected for inclusion in the validation set. - If the search strategy is to be peer reviewed, report the peer review process to be used, whether there is an aim to publish the review protocol (in which journal/section), and whether a specify any tool was used, such as the Peer Review of Electronic Search Strategies [PRESS (2015)](file:///C:\Users\jafonso\Desktop\PRESS%202015%20Guideline%20Evidence-Based%20Checklist) checklist ([see table 1 within this](https://www.jclinepi.com/action/showPdf?pii=S0895-4356%2816%2900058-5)). - If the search strategy structure adopted is not based on a PEO-style approach, describe the final conceptual structure and any explorations that were undertaken to achieve it. | Keywords and phrases:   - **Outcome domain and measures:** econom*, impact*, effect*, benefit*, cost-effect*, cost-efficien* - **Disease/condition of interest:** AMU, AMR, antimicrobial, usage, use, resistance - **Population: livestock, animal, production, food** - **Geographical setting: n/a** - **Time-period:** n/a   I don’t know where to put intervent*, strategy* |
| RELEVANT PUBLICATIONS | 8 | - Provide hyperlinks (to Open Access sources) to publications already known to be highly relevant to the topic | - *Hyperlink* - *Hyperlink* |

# Search codes for identifying papers

## Ovid Medline (Ovid MEDLINE(R) ALL <1946 to July 06, 2022>)

1 animal health.tw. 10180

2 Animals, Domestic/ or Service Animals/ 16026

3 Livestock/ 4839

4 livestock.tw. 30659

5 Swine/ or Cattle/ or production animal*.tw. 567645

6 sheep.tw. or Sheep/ or Sheep, Domestic/ 149286

7 goat.tw. or Goats/ 42015

8 small ruminant*.tw. 3501

9 Buffaloes/ or large ruminant*.tw. 6928

10 Chickens/ or chicken*.tw. 169879

11 poultry.tw. or Poultry/ 39442

12 crustaceans.tw. or Crustacea/ or Fishes/ or fish*.tw. 295499

13 1 or 2 or 3 or 4 or 5 or 6 or 7 or 8 or 9 or 10 or 11 or 12 1215658

14 Anti-Infective Agents/ or Anti-Bacterial Agents/ or amu.tw. 427932

15 anti-microbial.tw. 4921

16 Drug Resistance, Microbial/ or antimicrobial resistance.tw. or Drug Resistance, Multiple, Bacterial/ or Drug Resistance, Bacterial/ 142223

17 antiviral*.tw. or Antiviral Agents/ 158345

18 antimicrobial us*.tw. 3984

19 Antifungal Agents/ 64161

20 14 or 15 or 16 or 17 or 18 or 19 705726

21 intervent*.tw. 1197217

22 strateg*.tw. 1337815

23 treatment*.tw. or Treatment Outcome/ 5623780

24 21 or 22 or 23 7221436

25 econom*.tw. or Economics/ 374618

26 impact*.tw. 1372897

27 effect*.tw. 7751657

28 benefit*.tw. 794973

29 cost-effect*.tw. 158111

30 cost-efficien*.tw. 9411

31 25 or 26 or 27 or 28 or 29 or 30 9191840

32 13 and 20 35362

33 24 and 31 3348795

34 32 and 33 5916

35 limit 34 to yr="2000 -Current" 5282

N=5282 papers were downloaded from OVID Medline.

## CABI (Abstracts and Global Health)

("Drug Resistance" OR "antibacterial" OR antiviral* OR "antimicrobial" OR Antifungal Agent* OR Anti-Infective Agent* OR Anti-Bacterial Agent* OR "AMU" OR "AMR" OR "antibiotics") AND (((econom* OR impact* OR effect* OR benefit* OR cost-effect* OR cost-efficien*)) AND (subject:(livestock or cattle OR zebu OR bovine OR goat* OR sheep OR small ruminant* OR ruminant* OR pig* OR swine OR chicken* OR ruminant* OR *livestock OR buffalo* OR poultry OR crustacea* OR fish*)))

Haven’t included as sample size low anyway. (intervent* OR strateg* OR treatment* or Treatment Outcome)

N=1018 were downloaded from CABI.

## Scopus

( TITLE-ABS-KEY ( econom* OR impact* OR effect* OR benefit* OR cost-effect* OR cost-efficien* ) ) AND ( ( TITLE-ABS-KEY ( livestock OR cattle OR zebu OR buffalo OR bovine OR goat* OR sheep OR small AND ruminant* OR ruminant* OR pig* OR poultry OR swine OR chicken* OR ruminant* OR *livestock OR crustacea* OR fish* ) ) AND ( TITLE-ABS-KEY ( drug AND resistance OR antibacterial OR antiviral* OR antimicrobial OR antifungal AND agent* OR anti-infective AND agent* OR anti-bacterial AND agent* OR "AMU" OR "AMR" OR antibiotics ) ) ) AND ( LIMIT-TO ( SUBJAREA , "MEDI" ) OR LIMIT-TO ( SUBJAREA , "VETE" ) OR LIMIT-TO ( SUBJAREA , "AGRI" ) OR LIMIT-TO ( SUBJAREA , "ENVI" ) OR LIMIT-TO ( SUBJAREA , "MULT" ) OR LIMIT-TO ( SUBJAREA , "COMP" ) OR LIMIT-TO ( SUBJAREA , "ECON" ) OR LIMIT-TO ( SUBJAREA , "MATH" ) OR LIMIT-TO ( SUBJAREA , "HEAL" ) ) AND ( LIMIT-TO ( PUBYEAR , 2022 ) OR LIMIT-TO ( PUBYEAR , 2021 ) OR LIMIT-TO ( PUBYEAR , 2020 ) OR LIMIT-TO ( PUBYEAR , 2019 ) OR LIMIT-TO ( PUBYEAR , 2018 ) OR LIMIT-TO ( PUBYEAR , 2017 ) OR LIMIT-TO ( PUBYEAR , 2016 ) OR LIMIT-TO ( PUBYEAR , 2015 ) OR LIMIT-TO ( PUBYEAR , 2014 ) OR LIMIT-TO ( PUBYEAR , 2013 ) OR LIMIT-TO ( PUBYEAR , 2012 ) OR LIMIT-TO ( PUBYEAR , 2011 ) OR LIMIT-TO ( PUBYEAR , 2010 ) OR LIMIT-TO ( PUBYEAR , 2009 ) OR LIMIT-TO ( PUBYEAR , 2008 ) OR LIMIT-TO ( PUBYEAR , 2007 ) OR LIMIT-TO ( PUBYEAR , 2006 ) OR LIMIT-TO ( PUBYEAR , 2005 ) OR LIMIT-TO ( PUBYEAR , 2004 ) OR LIMIT-TO ( PUBYEAR , 2003 ) OR LIMIT-TO ( PUBYEAR , 2002 ) OR LIMIT-TO ( PUBYEAR , 2001 ) OR LIMIT-TO ( PUBYEAR , 2000 ) )

N=2643 were downloaded from SCOPUS.

# List of references

Table S.2. References and their general characteristics

| Author/year | Study type | Country | AMs | Alternative | Study unit | No study Units | No farms | No animals* |
| --- | --- | --- | --- | --- | --- | --- | --- | --- |
| Beef Cattle |  |  |  |  |  |  |  |  |
| Becker, J. et al., 2020 | Prospective non-RCT | Switzerland | ABs | Farm Management | Calf | 1905 | 38 | n/a |
| Berman, J. et al., 2017 | Prospective RCT | Canada | ABs | No use of AMs | Calf | 209 | 1 | n/a |
| Catry, B. et al., 2008 | Prospective RCT | The Netherlands | ABs | No use of AMs | Calf | 695 | 1 | n/a |
| Cusack, P. M. V., 2004 | Prospective RCT | Australia | ABs | No use of AMs | Cattle | 630 | 1 | n/a |
| Davedow, T. et al., 2020 | Prospective RCT | Canada | ABs | New therapy protocol | Cattle And Pen | 7576 | 1 | n/a |
| Diana, A. et al., 2022 | Prospective non-RCT | Italy | ABs | Farm Management | Bull | 576 | 5 | n/a |
| Dorado-Garcia, A. et al., 2015 | Prospective RCT | The Netherlands | ABs | Multiple | Farm | n/a | 51 | n/a |
| Godinho, K. S. et al., 2005 | Prospective RCT | Multiple | ABs | No use of AMs | Cattle | 1251 | no info | n/a |
| Hendrick, S. H. et al., 2013 | Prospective RCT | Canada | ABs | No use of AMs | Calf | 3784 | 1 | n/a |
| Hibbard, B. et al., 2002 | Prospective RCT | USA | ABs | No use of AMs | Cattle | 4073 | 10 | n/a |
| Misawa, N. et al., 2000 | Pre-post intervention study | Japan | ABs | No use of AMs | Calf | 30 | 3 | n/a |
| O'Connor, A. et al., 2001 | Prospective non-RCT | Canada | ABs | Vaccines | Calf | 852 | 3 | n/a |
| Rae, D. O. et al., 2002 | Prospective RCT | USA | ABs | No use of AMs | Heifer | 768 | 1 | n/a |
| Regev-Shoshani, G. et al., 2017 | Prospective RCT | Canada | ABs | Other | Calf | 1080 | 1 | n/a |
| Santinello, M. et al., 2022 | Prospective non-RCT | Italy | ABs | Farm Management | Cattle | 1206 | 5 | n/a |
| Senturk, S. et al., 2007 | Prospective RCT | Turkey | ABs | No use of AMs | Cow | 36 | no info | n/a |
| Smith, A. B. et al., 2017 | Prospective RCT | USA | ABs | No use of AMs | Calf | 298 | 1 | n/a |
| Tennant, T. C. et al., 2014 | Prospective RCT | USA | ABs | No use of AMs | Cattle | 2336 | 1 | n/a |
| Zielinski, G. C. et al., 2002 | Prospective RCT | Argentina | ABs | New therapy protocol | Cattle | 120 | 1 | n/a |
| Broilers |  |  |  |  |  |  |  |  |
| Bailey, M. A. et al., 2019 | Prospective observational study | USA | No AMs used | No use of AMs | Sample | 1232 | no info | no info |
| Bugener, E. et al., 2014 | Prospective non-RCT | Germany | ABs | Feed/Water Management | Flock | 6 | 2 | no info |
| Caekebeke, N. et al., 2021 | Pre-post intervention study | Multiple | ABs | Animal Health Advisory/Training | Farm | n/a | 30 | n/a |
| Debnam, A. L. et al., 2005 | Prospective non-RCT | USA | ABs | No use of AMs | Sample | 1478 | 1 | no info |
| Garces-Narro, C. et al., 2013 | Prospective RCT | Spain | ABs | No use of AMs | Pen | 24 | 1 | 1440 |
| Parent, E. et al., 2020 | Prospective RCT | Canada | ABs | Prebiotic | Flock | 84 | 7 | no info |
| Pedroso, A. A. et al., 2013 | Prospective RCT | USA | ABs | Multiple | Flock | 60 | 3 | no info |
| Roskam, J. L. et al., 2019 | Pre-post intervention study | Unknown | ABs | Animal Health Advisory/Training | Farm | n/a | 20 | n/a |
| Smialek, M. et al., 2020 | Prospective non-RCT | Poland | ABs | Vaccines | Flock | 6 | 2 | no info |
| Turcotte, C. et al., 2020 | Prospective non-RCT | Canada | ABs | Farm Management | Chicken | 288 | 6 | n/a |
| Dairy cattle |  |  |  |  |  |  |  |  |
| Absalon-Medina, V. A. et al., 2022 | Prospective RCT | USA | ABs | No use of AMs | Heifer | 132 | 1 | n/a |
| Afema, J. A. et al., 2019 | Pre-post intervention study | USA | ABs | New therapy protocol | Calf | 4301 | 1 | n/a |
| Barlow, J. W. et al., 2013 | Prospective RCT | USA | ABs | No use of AMs | Mamary Quarter | 1492 | 2 | 373 |
| Bartolome, J. A. et al., 2014 | Prospective RCT | Argentina | ABs | New therapy protocol | Cow | 1104 | 1 | n/a |
| Bates, A. et al., 2020 | Prospective RCT | New Zealand | ABs | New therapy protocol | Mamary Quarter | 648 | 7 | 608 |
| Beggs, D. S. and Wraight, M. D., 2006 | Prospective non-RCT | Australia | ABs | No use of AMs | Cow | 69 | 5 | n/a |
| Berge, A. C. B. et al., 2009b | Prospective RCT | USA | ABs | New therapy protocol | Calf | 358 | 1 | n/a |
| Berry, E. A. and Hillerton, J. E., 2002a | Prospective RCT | UK | ABs | No use of AMs | Mamary Quarter | 1258 | 4 | 290 |
| Berry, E. A. and Hillerton, J. E., 2002b | Prospective RCT | Ireland | No AMs used | New therapy protocol | Cow | 398 | 7 | n/a |
| Bhutto, A. L. et al., 2011 | Prospective RCT | UK | ABs | New therapy protocol | Mamary Quarter | 960 | 2 | 240 |
| Binversie, E. S. et al., 2020 | Prospective RCT | USA | ABs | No use of AMs | Calf | 357 | 2 | n/a |
| Bradley, A. J. et al., 2010 | Prospective RCT | UK | ABs | New therapy protocol | Mamary Quarter | 1620 | 6 | 810 |
| Brick, T. A. et al., 2012 | Prospective RCT | USA | ABs | Other | Cow | 237 | 2 | n/a |
| Bryan, M. and Taylor, K., 2009 | Prospective RCT | New Zealand | ABs | No use of AMs | Heifer | 598 | 3 | n/a |
| Cameron, M. et al., 2014 | Prospective RCT | Canada | ABs | New therapy protocol | Mamary Quarter | 2287 | 16 | 603 |
| Cameron, M. et al., 2015 | Prospective RCT | Canada | ABs | New therapy protocol | Cow | 600 | 16 | n/a |
| Cao, L. T. et al., 2007 | Prospective RCT | China | ABs | Bioactive Protein And Peptides | Mamary Quarter | 107 | 1 | 92 |
| Catozzi, C. et al., 2019 | Prospective non-RCT | Italy | ABs | Probiotics | Mamary Quarter | 43 | 1 | 20 |
| Clabby, C. et al., 2022 | Prospective RCT | Ireland | ABs | New therapy protocol | Mamary Quarter And Cow | 2981 | 5 | 842 |
| de Oliveira, E. B. et al., 2020 | Prospective RCT | USA | ABs | Prebiotic | Cow | 826 | 3 | n/a |
| Denis-Robichaud, J. and Dubuc, J., 2015 | Prospective RCT | Canada | ABs | No use of AMs | Cow | 2259 | 28 | n/a |
| Drillich, M. et al., 2006 | Prospective non-RCT | Germany | ABs | Other | Cow | 501 | 5 | n/a |
| Dubuc, J. et al., 2011 | Prospective RCT | Canada | ABs | No use of AMs | Cow | 2178 | 6 | n/a |
| Elmetwally, M. A. et al., 2020 | Prospective non-RCT | Egypt | ABs | New therapy protocol | Buffalo | 65 | no info | n/a |
| Elsener, J. et al., 2001 | Prospective RCT | Canada | AHs | No use of AMs | Heifer | 84 | 1 | n/a |
| Fuenzalida, M. J. and Ruegg, P. L., 2019 | Prospective RCT | USA | ABs | No use of AMs | Cow | 168 | 2 | n/a |
| Fuenzalida, M. J. and Ruegg, P. L., 2019 | Prospective RCT | USA | ABs | No use of AMs | Cow | 194 | 2 | n/a |
| Galvao, K. N. et al., 2009 | Prospective RCT | USA | ABs | No use of AMs | Cow | 812 | 1 | n/a |
| Galvao, K. N. et al., 2020 | Prospective RCT | USA | ABs | Prebiotic | Cow | 89 | 1 | n/a |
| Giuliodori, M. J. et al., 2013 | Prospective RCT | Argentina | ABs | No use of AMs | Cow | 303 | 1 | n/a |
| Godden, S. et al., 2003 | Prospective RCT | USA | ABs | New therapy protocol | Mamary Quarter | 1748 | 2 | 437 |
| Gomez, D. E. et al., 2017 | Pre-post intervention study | Canada | ABs | New therapy protocol | Calf | 2649 | 2 | n/a |
| Gomez, D. E. et al., 2021 | Pre-post intervention study | Canada | ABs | Multiple | Calf | 4300 | 10 | n/a |
| Goshen, T. and Shpigel, N. Y.., 2006 | Prospective RCT | Israel | ABs | No use of AMs | Cow | 2220 | 5 | n/a |
| Griffioen, K. et al., 2021 | Prospective RCT | The Netherlands | ABs | New therapy protocol | Cow | 233 | 15 | n/a |
| Guccione, J. et al., 2014 | Prospective RCT | Italy | ABs | No use of AMs | Buffalo | 40 | 1 | n/a |
| Guccione, J. et al., 2020 | Prospective RCT | Italy | ABs | No use of AMs | Mamary Quarter | 160 | 1 | 40 |
| Hallberg, J. W. et al., 2006 | Prospective RCT | USA | ABs | No use of AMs | Mamary Quarter | 1769 | 21 | 431 |
| Hektoen, L. et al., 2004 | Prospective RCT | Norway | ABs | Other | Cow | 57 | 39 | n/a |
| Hendrick, S. H. et al., 2006 | Prospective RCT | Canada | ABs | No use of AMs | Cow | 233 | 13 | n/a |
| Jacobs, C. et al., 2018 | Prospective RCT | Canada | ABs | New therapy protocol | Hoof | 1372 | 10 | 1070 |
| Jeremejeva, J. et al., 2012 | Prospective RCT | Estonia | ABs | New therapy protocol | Cow And Sample | 201 | 1 | 51 |
| Joachim, A. et al., 2003 | Prospective RCT | Germany | ABs | Other | Calf | 152 | 7 | n/a |
| Kabera, F. et al., 2020 | Prospective RCT | Canada | ABs | New therapy protocol | Mamary Quarter | 2247 | 9 | 568 |
| Kai, K. et al., 2002 | Prospective RCT | Japan | ABs | Bioactive Protein And Peptides | Mamary Quarter | 41 | 3 | 36 |
| Kaithwas, G. et al., 2011 | Prospective non-RCT | India | ABs | Plant-Based (Phytogenic) | Cow | 9 | 2 | n/a |
| Kaneene, J. B. et al., 2008 | Prospective non-RCT | USA | ABs | Feed/Diet Management | Sample And Isolate | 1724 | 8 | 2763 |
| Kasimanickam, R. et al., 2005 | Prospective RCT | Canada | ABs | New therapy protocol | Cow | 228 | 2 | n/a |
| Kasravi, R. et al., 2011 | Prospective RCT | Iran | ABs | No use of AMs | Mamary Quarter | 150 | 1 | 73 |
| Keller, D. and Sundrum, A., 2018 | Prospective RCT | Germany | ABs | Other | Mamary Quarter | 180 | 4 | 180 |
| Knutti, B. et al., 2000 | Prospective observational study | Switzerland | ABs | New therapy protocol | Lactation | 6598 | 85 | 3276 |
| Kreiger, M. et al., 2007 | Prospective RCT | Austria | ABs | No use of AMs | Cow | 59 | 7 | n/a |
| Kuipers, A. et al., 2016 | Pre-post intervention study | The Netherlands | ABs | Animal Health Advisory/Training | Farm | 10480 | 94 | n/a |
| Lago, A. et al., 2011 | Prospective RCT | Multiple | ABs | New therapy protocol | Mamary Quarter | 449 | 8 | 422 |
| Lago, A. et al., 2011 | Prospective RCT | Multiple | ABs | New therapy protocol | Mamary Quarter And Cow | 449 | 8 | 422 |
| Laven, R. A. and Hunt, H., 2002 | Prospective RCT | UK | ABs | Farm Management | Lesion | 252 | 1 | 169 |
| LeBlanc, S. J. et al., 2002 | Prospective RCT | Canada | ABs | New therapy protocol | Cow | 316 | 27 | n/a |
| Leitner, G. et al., 2018 | Prospective non-RCT | Israel | ABs | Other | Mamary Quarter And Cow | 145 | 3 | 145 |
| Lendzele, S. S. et al., 2021 | Prospective non-RCT | Cameroon | ABs | New therapy protocol | Cattle | 36 | 12 | n/a |
| Machado, V. S. and Bicalho, R. C., 2018 | Prospective RCT | USA | ABs | New therapy protocol | Heifer | 886 | 1 | n/a |
| Madoz, L. V. et al., 2017 | Prospective RCT | Multiple | ABs | New therapy protocol | Cow | 352 | 2 | n/a |
| Mahendran, S. A. et al., 2017 | Prospective RCT | UK | ABs | New therapy protocol | Calf | 154 | 2 | n/a |
| Malinowski, E. et al., 2019 | Prospective non-RCT | Poland | ABs | Other | Mamary Quarter And Cow | 167 | 1 | 124 |
| Manske, T. et al., 2002 | Prospective quasi-RCT | Sweden | ABs | New therapy protocol | Hoof | 224 | 1 | 56 |
| Mari, G. et al., 2012 | Prospective RCT | Italy | ABs | No use of AMs | Cow | 80 | 2 | n/a |
| McDougall, S. et al., 2013 | Prospective RCT | New Zealand | ABs | New therapy protocol | Cow | 756 | 36 | n/a |
| McDougall, S. et al., 2022 | Prospective RCT | New Zealand | ABs | New therapy protocol | Mamary Quarter And Cow | 6528 | 4 | 1632 |
| Mullen, K. A. et al., 2014 | Prospective non-RCT | USA | ABs | Plant-Based (Phytogenic) | Mamary Quarter | 1044 | 5 | 441 |
| Orjales, I. et al., 2016 | Prospective observational study | Spain | ABs | No use of AMs | Farm | 393 | 18 | n/a |
| Passchyn, P. et al., 2013 | Prospective non-RCT | Belgium | ABs | No use of AMs | Heifer | 149 | 10 | n/a |
| Pempek, J. A. et al., 2019 | Prospective RCT | USA | ABs | Bioactive Protein And Peptides | Calf | 485 | 5 | n/a |
| Pereira, R. V. et al., 2020 | Prospective RCT | USA | ABs | No use of AMs | Calf | 65 | 1 | n/a |
| Pinedo, P. J. et al., 2015 | Prospective RCT | USA | No AMs used | Other | Cow | 220 | 1 | n/a |
| Rajala-Schultz, P. J. et al., 2011 | Prospective RCT | USA | ABs | New therapy protocol | Cow | 723 | 4 | n/a |
| Raymond, M. J. et al., 2006 | Pre-post intervention study | USA | ABs | Animal Health Advisory/Training | Farm | n/a | 292 | n/a |
| Relun, A. et al., 2013 | Prospective RCT | France | Other | Farm Management | Feet | 5598 | 52 | 4678 |
| Rowe, S. M. et al., 2020 | Prospective RCT | USA | ABs | New therapy protocol | Mamary Quarter | 4173 | 7 | 1176 |
| Rowe, S. M. et al., 2020 | Prospective RCT | USA | ABs | New therapy protocol | Cow | 1211 | 7 | n/a |
| Roy, J. et al., 2007 | Prospective non-RCT | Canada | ABs | No use of AMs | Heifer | 428 | 23 | n/a |
| Roy, J. et al., 2009 | Prospective RCT | Canada | ABs | No use of AMs | Cow | 61 | 14 | n/a |
| Runciman, D. J. et al., 2008 | Prospective non-RCT | Australia | ABs | No use of AMs | Cow | 1325 | 17 | n/a |
| Salat, O. et al., 2008 | Prospective RCT | France | ABs | No use of AMs | Mamary Quarter | 151 | 53 | 92 |
| Sampimon, O. C. et al., 2009 | Prospective RCT | The Netherlands | ABs | No use of AMs | Heifer | 392 | 13 | n/a |
| Sandgren, C. H. et al., 2008 | Prospective RCT | Sweden | ABs | New therapy protocol | Cow | 126 | 20 | n/a |
| Sannmann, I. et al., 2013 | Prospective RCT | Germany | ABs | New therapy protocol | Cow | 193 | 1 | n/a |
| Santman-Berends, I. M. G. A. et al., 2021 | Pre-post intervention study | the Netherlands | ABs | Regulation | Cow | 1686168 | 17032 | n/a |
| Schmenger, A. et al., 2020 | Prospective non-RCT | Germany | ABs | New therapy protocol | Cow | 1392 | 5 | n/a |
| Schultz, N. and Capion, N., 2013 | Prospective RCT | Denmark | ABs | New therapy protocol | Cow | 54 | 1 | n/a |
| Sellera, F. P. et al., 2021 | Prospective RCT | Brazil | ABs | Other | Hoof | 20 | 2 | 16 |
| Shearer, J. K. and Hernandez, J.., 2000 | Prospective RCT | USA | ABs | New therapy protocol | Cow | 65 | 1 | n/a |
| Shephard, R. W. et al., 2000 | Prospective RCT | Australia | ABs | No use of AMs | Cow | 438 | 35 | n/a |
| Shim, E. H. et al., 2004 | Prospective RCT | USA | ABs | New therapy protocol | Lactation | 124 | 1 | no info |
| Shinozuka, Y. et al., 2009 | Prospective RCT | Japan | ABs | New therapy protocol | Cow | 57 | 30 | n/a |
| Silva, L. A. F. et al, 2005 | Prospective RCT | Brazil | ABs | Multiple | Cow | 120 | 2 | n/a |
| Speksnijder, D. C. et al., 2017 | Prospective RCT | the Netherlands | ABs | Animal Health Advisory/Training | Farm | n/a | 39 | n/a |
| St Rose, S. G. et al., 2003 | Prospective RCT | The Netherlands | ABs | No use of AMs | Mamary Quarter | 58 | 39 | 55 |
| Steele, N. and McDougall, S., 2014 | Prospective non-RCT | New Zealand | ABs | No use of AMs | Mamary Quarter | 178 | 3 | 92 |
| Stevens, M. et al., 2019 | Prospective RCT | Belgium | No AMs used | Animal Health Advisory/Training | Farm | n/a | 56 | n/a |
| Suojala, L. et al., 2010 | Prospective RCT | Finland | ABs | No use of AMs | Cow | 132 | 61 | n/a |
| Tartor, Y. H. et al., 2020 | Prospective RCT | Egypt | AFs | Plant-Based (Phytogenic) | Calf | 75 | no info | n/a |
| Taurel, A. et al., 2012 | Prospective RCT | France | ABs | Vaccines | Cow | 883 | 22 | n/a |
| Teixeira, A. G. V. et al., 2017 | Prospective RCT | USA | ABs | No use of AMs | Calf | 795 | 1 | n/a |
| Tetens, J. L. et al., 2019 | Prospective observational study | Germany | ABs | New therapy protocol | Calf | 50 | 2 | n/a |
| Tison, N. et al., 2017 | Prospective RCT | Canada | ABs | No use of AMs | Cow | 1247 | 18 | n/a |
| Tomazi, T. et al., 2021 | Prospective RCT | USA | ABs | No use of AMs | Mamary Quarter | 696 | 1 | 585 |
| van den Borne, B. H. P. et al., 2019 | Prospective RCT | The Netherlands | ABs | No use of AMs | Mamary Quarter | 634 | 38 | 486 |
| Vasquez, A. K. et al., 2015 | Prospective RCT | USA | ABs | New therapy protocol | Cow | 489 | 1 | n/a |
| Vasquez, A. K. et al., 2018 | Prospective RCT | USA | ABs | New therapy protocol | Cow And Mamary Quarter | 2098 | 1 | 574 |
| Werner, C. et al., 2010 | Prospective RCT | Germany | ABs | Other | Mamary Quarter | 147 | 4 | 136 |
| Whist, A. C. et al., 2006 | Prospective RCT | Norway | ABs | New therapy protocol | Lactation | 8229 | 164 | 4557 |
| Wittek, T. et al., 2018 | Retrospective observational study | Austria | ABs | No use of AMs | Lactation | 88534 | 1657 | 88534 |
| Woolford, M. W. et al., 2001 | Prospective RCT | New Zealand | ABs | New therapy protocol | Mamary Quarter | 2493 | 3 | 632 |
| Zobel, R. and Tkalcic, S., 2013 | Prospective non-RCT | Croatia | ABs | Other | Cow | 400 | 2 | n/a |
| Goats |  |  |  |  |  |  |  |  |
| McDougall, S. et al., 2010 | Prospective RCT | New Zealand | ABs | No use of AMs | Mamary Gland | 106 | 4 | 52 |
| Layers |  |  |  |  |  |  |  |  |
| Caudell, M. A. et al., 2022 | Prospective non-RCT | Multiple | ABs | Animal Health Advisory/Training | Farmer | n/a | no info | n/a |
| Ramirez, S. Y. et al., 2021 | Prospective RCT | Colombia | ABs | Plant-Based (Phytogenic) | Laying Hen | 96 | 1 | n/a |
| Schwaiger, K. et al., 2010 | Prospective observational study | Germany | ABs | Farm Management | Sample | 1599 | 20 | 800 |
| Multiple |  |  |  |  |  |  |  |  |
| Berge, A. C. B. et al., 2009a | Prospective RCT | USA | ABs | Feed/Diet Management | Calf | 273 | 3 | n/a |
| Jabbar, A. et al., 2022 | Prospective RCT | Pakistan | AHs | Plant-Based (Phytogenic) | Small Ruminant | 720 | no info | n/a |
| Kaiaty, A. M. et al., 2021 | Prospective RCT | Egypt | AHs | Plant-Based (Phytogenic) | Livestock | 120 | no info | n/a |
| Lhermie, G. et al., 2017 | Prospective RCT | France | ABs | No use of AMs | Cattle And Sample | 162 | 6 | 78 |
| Sheep |  |  |  |  |  |  |  |  |
| Astobiza, I. et al., 2013 | Prospective non-RCT | Spain | ABs | Multiple | Ewe | 81 | 1 | n/a |
| Croft, A. et al., 2000 | Prospective RCT | Canada | ABs | No use of AMs | Ewe And Lamb | 2084 | 9 | n/a |
| Duncan, J. S. et al., 2012 | Prospective RCT | UK | ABs | Vaccines | Lamb | 748 | 1 | n/a |
| Gonzalo, C. et al., 2004 | Prospective RCT | Spain | ABs | No use of AMs | Half Udder | 566 | 1 | 286 |
| Hernandez, F. et al., 2015 | Pre-post intervention study | Spain | ABs | No use of AMs | Lactation | 5981 | 1 | 1951 |
| Holsback, L. et al., 2016 | Prospective RCT | Brazil | AHs | No use of AMs | Ewe | 180 | 1 | n/a |
| Kaler, J. et al., 2010 | Prospective RCT | UK | ABs | Multiple | Sheep | 53 | 1 | n/a |
| Learmount, J. et al., 2015 | Prospective non-RCT | UK | AHs | New therapy protocol | Lamb And Ewe | 900 | 16 | n/a |
| Learmount, J. et al., 2016 | Prospective non-RCT | UK | AHs | New therapy protocol | Farm | n/a | 16 | n/a |
| Maingi, N. et al., 2002 | Prospective RCT | Kenya | AHs | No use of AMs | Lamb | 30 | 1 | n/a |
| Mugnaini, L. et al., 2013 | Prospective RCT | Italy | No AMs used | Plant-Based (Phytogenic) | Sheep | 22 | 1 | n/a |
| Rizzon Cintra, M. C. et al., 2019 | Prospective non-RCT | Brazil | AHs | New therapy protocol | Lamb | 118 | 2 | n/a |
| Swine |  |  |  |  |  |  |  |  |
| Abubakar, R. H. et al., 2019 | Prospective RCT | South Africa | ABs | No use of AMs | Sample | 320 | 1 | 10 |
| Alexopoulos, C. et al., 2006 | Prospective RCT | Greece | ABs | No use of AMs | Pig | 240 | 1 | n/a |
| Arkfeld, E. K. et al., 2015 | Prospective RCT | USA | ABs | No use of AMs | Pig | 1232 | no info | n/a |
| Byra, C. et al., 2011 | Prospective RCT | Canada | ABs | No use of AMs | Pig | 896 | 1 | n/a |
| Cameron-Veas, K. et al., 2016 | Prospective RCT | Spain | ABs | No use of AMs | Pig | 560 | 8 | n/a |
| Che, T. M. et al., 2012 | Prospective RCT | USA | ABs | Feed/Diet Management | Pig | 1008 | 1 | n/a |
| Chekabab, S. M. et al., 2020 | Prospective observational study | Canada | ABs | No use of AMs | Sample | 72 | 4 | no info |
| Choi, SH. and Kang, SS., 2001 | Prospective RCT | Republic of Korea | ABs | Other | Sample | 42 | no info | 42 |
| Collineau, L. et al., 2017 | Pre-post intervention study | Multiple | ABs | Animal Health Advisory/Training | Farm | n/a | 68 | n/a |
| Correa-Fiz, F. et al., 2019 | Pre-post intervention study | Spain | ABs | No use of AMs | Sample | no info | 2 | no info |
| Correa-Fiz, F. et al., 2020 | Prospective RCT | Spain | ABs | Multiple | Piglets | 569 | 1 | n/a |
| Cremonesi, P. et al., 2022 | Prospective RCT | Italy | ABs | Plant-Based (Phytogenic) | Piglets | 197 | 1 | n/a |
| De Lucia, A. et al., 2021 | Pre-post intervention study | UK | ABs | No use of AMs | Bacteria Isolate | 259 | 1 | no info |
| Diana, A. et al., 2017 | Prospective RCT | Ireland | ABs | No use of AMs | Pig | 840 | 1 | n/a |
| Diana, A. et al., 2019 | Prospective RCT | Ireland | ABs | Regulation | Pig | 840 | 1 | n/a |
| Dohmen, W. et al., 2017 | Pre-post intervention study | The Netherlands | ABs | Animal Health Advisory/Training | Farm And Sample | 1350 | 36 | 8640 |
| Dorado-García, A. et al., 2015 | Pre-post intervention study | the Netherlands | ABs | Animal Health Advisory/Training | Farm | n/a | 36 | n/a |
| Duggett, N. A. et al., 2018 | Pre-post intervention study | UK | ABs | No use of AMs | Sample | 349 | 1 | no info |
| Funk, J. et al., 2007 | Prospective RCT | USA | ABs | Farm Management | Sample | 1800 | 1 | no info |
| Giannakopoulos, C. G. et al., 2001 | Prospective RCT | Greece | ABs | No use of AMs | Gilt And Sow | 250 | 1 | n/a |
| Heller, O. et al., 2016 | Prospective observational study | Switzerland | ABs | No use of AMs | Sample | 268 | 27 | no info |
| Jensen, K. J. et al., 2022 | Prospective RCT | Denmark | ABs | Vaccines | Sow And Piglet | 6322 | 1 | n/a |
| Kaiser, M. et al., 2013 | Prospective RCT | Denmark | ABs | Other | Lesion | 299 | 3 | 304 |
| Kyriakis, S. C. et al., 2002 | Prospective RCT | Greece | ABs | No use of AMs | Piglets | 288 | 1 | n/a |
| Kyriakis, S. C. et al., 2002 | Prospective RCT | Greece | ABs | No use of AMs | Piglets | 288 | 1 | n/a |
| Laine, T. et al., 2004 | Pre-post intervention study | Finland | ABs | Regulation | Farm | n/a | 73 | n/a |
| Mateusen, B. et al., 2001 | Prospective RCT | Belgium | ABs | Vaccines | Piglets | 204 | 1 | n/a |
| Mateusen, B. et al., 2002 | Prospective RCT | Belgium | ABs | Vaccines | Piglets | 245 | 1 | n/a |
| Menezes, T. A. et al., 2020 | Prospective non-RCT | Brazil | ABs | Bioactive Protein And Peptides | Sample | 204 | 1 | 34 |
| Peng, Z. et al., 2016 | Prospective RCT | China | ABs | Animal Health Advisory/Training | Piglets | 120 | 1 | n/a |
| Postma, M. et al., 2017 | Pre-post intervention study | Belgium | ABs | Animal Health Advisory/Training | Farm | n/a | 61 | n/a |
| Raasch, S. et al., 2020 | Pre-post intervention study | Multiple | ABs | Prebiotic | Farm | n/a | 68 | n/a |
| Rozeboom, D. W. et al., 2005 | Prospective RCT | USA | ABs | Animal Health Advisory/Training | Pig | 1443 | 3 | n/a |
| Shen, L. et al., 2021 | Pre-post intervention study | China | ABs | Vaccines | Farmer | no info | no info | n/a |
| Sjolund, M. and Wallgren, P., 2010 | Pre-post intervention study | Sweden | ABs | Animal Health Advisory/Training | Batch | 54 | 1 | no info |
| Toya, R. et al., 2022 | Prospective non-RCT | Japan | ABs | Regulation | Farm | n/a | 16 | n/a |
| van Asseldonk, M. et al., 2020 | Pre-post intervention study | the Netherlands | ABs | Regulation | Farm | n/a | variable | n/a |
| Vigre, H. et al., 2008 | Non-randomised case-crossover study | Denmark | ABs | No use of AMs | Pig Days And Farm | n/a | 68 | n/a |
| Yun, J. et al., 2017 | Prospective non-RCT | Finland | ABs | No use of AMs | Piglets | 7156 | 1 | n/a |
| Tilapia |  |  |  |  |  |  |  |  |
| Monteiro, S. H. et al., 2016 | Prospective observational study | Brazil | ABs | Farm Management | Fish | 126 | 4 | n/a |
| Turkey |  |  |  |  |  |  |  |  |
| Van Droogenbroeck, C. et al., 2011 | Prospective non-RCT | Belgium | No AMs used | Bioactive Protein And Peptides | Turkey | 11650 | 1 | n/a |

Table S.3. Qualitative assessment of the impact of the alternatives/interventions on the indicators across the references (+ means positive, - means negative, <> means bidirectional, = means equivalent)

| Author/year | AMs | Study disease(s) | Alternative | Indicator | | | | | | |
| --- | --- | --- | --- | --- | --- | --- | --- | --- | --- | --- |
|  |  |  |  | Production | AMU | Economic performance | AMR | Clinical | Epidemiology | Productquality |
| Beef Cattle |  |  |  |  |  |  |  |  |  |  |
| Becker, J. et al., 2020 | ABs |  | Farm Management | = | + |  |  |  | + |  |
| Berman, J. et al., 2017 | ABs | Bovine respiratory disease (BRD) | No use of AMs | = |  |  |  |  | = |  |
| Catry, B. et al., 2008 | ABs | Bovine respiratory disease (BRD) | No use of AMs | - |  |  |  | - |  |  |
| Cusack, P. M. V., 2004 | ABs | Bovine respiratory disease (BRD) | No use of AMs | - |  |  |  |  | - |  |
| Davedow, T. et al., 2020 | ABs | Liver abcess | New therapy protocol | - |  |  | = | = | = |  |
| Diana, A. et al., 2022 | ABs | Multiple | Farm Management | + | + |  |  |  |  |  |
| Dorado-Garcia, A. et al., 2015 | ABs | no info | Animal Health Advisory/Training | = | + |  |  |  | = |  |
| Godinho, K. S. et al., 2005 | ABs | bovine respiratory disease (BRD) | No use of AMs | - |  |  |  | - | - |  |
| Hendrick, S. H. et al., 2013 | ABs | bovine respiratory disease (BRD) | No use of AMs | = |  |  |  |  | + |  |
| Hibbard, B. et al., 2002 | ABs | bovine respiratory disease (BRD) | No use of AMs | - |  |  |  |  | - |  |
| Misawa, N. et al., 2000 | ABs | diarrhoea | No use of AMs |  |  |  |  | - |  |  |
| O'Connor, A. et al., 2001 | ABs | undifferentiated bovine respiratory disease (UBRD) | Vaccines |  |  |  |  |  | + |  |
| Rae, D. O. et al., 2002 | ABs | no info | No use of AMs | - |  |  |  |  |  |  |
| Regev-Shoshani, G. et al., 2017 | ABs | Undifferentiated fever, bovine respiratory disease complex (BRDc) | Other | = |  |  |  | = | = |  |
| Santinello, M. et al., 2022 | ABs | Multiple | Farm Management | + | + |  |  |  |  |  |
| Senturk, S. et al., 2007 | ABs | infectious bovine keratoconjunctivitis | No use of AMs |  |  |  |  | - |  |  |
| Smith, A. B. et al., 2017 | ABs | bovine respiratory disease (BRD) | No use of AMs |  |  |  | = | = |  |  |
| Tennant, T. C. et al., 2014 | ABs | bovine respiratory disease (BRD) | No use of AMs | - |  | - |  |  | - | - |
| Zielinski, G. C. et al., 2002 | ABs | infectious bovine keratoconjunctivitis (IBK) | New therapy protocol |  |  |  |  | - |  |  |
| Broilers |  |  |  |  |  |  |  |  |  |  |
| Bailey, M. A. et al., 2019 | No AMs used | Campylobacteriosis | No use of AMs |  |  |  | = |  | + |  |
| Bugener, E. et al., 2014 | ABs | no info | Water Management | = | + |  |  | + | + |  |
| Caekebeke, N. et al., 2021 | ABs | no info | Animal Health Advisory/Training | + | = |  |  |  |  |  |
| Debnam, A. L. et al., 2005 | ABs |  | No use of AMs |  |  |  |  |  | = |  |
| Garces-Narro, C. et al., 2013 | ABs | necrotic enteritis | No use of AMs | - |  |  |  |  | = |  |
| Parent, E. et al., 2020 | ABs | Multiple | Prebiotic | = |  |  |  | = |  |  |
| Pedroso, A. A. et al., 2013 | ABs | no info | Multiple |  |  |  | = |  | + |  |
| Roskam, J. L. et al., 2019 | ABs | no info | Animal Health Advisory/Training | <> | + | <> |  |  | - |  |
| Smialek, M. et al., 2020 | ABs | colibacillosis | Vaccines | + | + | + | + | + |  |  |
| Turcotte, C. et al., 2020 | ABs | necrotic enteritis | Farm Management |  |  |  | + |  |  |  |
| Dairy cattle |  |  |  |  |  |  |  |  |  |  |
| Absalon-Medina, V. A. et al., 2022 | ABs | spontaneous abortions | No use of AMs | = |  |  |  |  |  |  |
| Afema, J. A. et al., 2019 | ABs | Multiple | New therapy protocol |  |  |  | + |  |  |  |
| Barlow, J. W. et al., 2013 | ABs | mastitis (clinical and subclinical) | No use of AMs |  |  |  |  | - | - |  |
| Bartolome, J. A. et al., 2014 | ABs | Metritis and endometritis | New therapy protocol | = |  |  |  |  | - |  |
| Bates, A. et al., 2020 | ABs | Clinical mastitis | New therapy protocol | = | + |  |  | = |  | = |
| Beggs, D. S. and Wraight, M. D., 2006 | ABs | Subclinical mastitis | No use of AMs |  |  |  |  |  |  | - |
| Berge, A. C. B. et al., 2009b | ABs | Multiple | New therapy protocol | + |  | + |  |  | + |  |
| Berry, E. A. and Hillerton, J. E., 2002a | ABs | Clinical mastitis | No use of AMs |  |  |  |  |  | - |  |
| Berry, E. A. and Hillerton, J. E., 2002b | No AMs used | Clinical mastitis | New therapy protocol |  |  |  |  |  | - |  |
| Bhutto, A. L. et al., 2011 | ABs | Clinical mastitis | New therapy protocol |  |  |  |  |  | = |  |
| Binversie, E. S. et al., 2020 | ABs | bovine respiratory disease (brd) | No use of AMs | + |  |  |  |  | - |  |
| Bradley, A. J. et al., 2010 | ABs | Clinical mastitis | New therapy protocol |  |  |  |  | - | - |  |
| Brick, T. A. et al., 2012 | ABs | Clinical endometritis | Other | = |  |  |  | = | = |  |
| Bryan, M. and Taylor, K., 2009 | ABs | Clinical mastitis | No use of AMs |  |  |  |  | - | - |  |
| Cameron, M. et al., 2014 | ABs | Clinical mastitis | New therapy protocol |  | + |  |  | = | = |  |
| Cameron, M. et al., 2015 | ABs | Clinical mastitis | Farm Management | = |  |  |  |  |  | = |
| Cao, L. T. et al., 2007 | ABs | Clinical mastitis | Bioactive Protein And Peptides |  |  |  | + | = |  | = |
| Catozzi, C. et al., 2019 | ABs | subclinical mastitis | Probiotics |  |  |  |  | - |  |  |
| Clabby, C. et al., 2022 | ABs | Clinical mastitis | New therapy protocol | = |  |  |  |  | - | - |
| de Oliveira, E. B. et al., 2020 | ABs | Metritis | Prebiotic | - |  |  |  | - | - |  |
| Denis-Robichaud, J. and Dubuc, J., 2015 | ABs | metritis | No use of AMs | - |  |  |  |  |  |  |
| Drillich, M. et al., 2006 | ABs | retained fetal membranes (RFM) | Other | = | - |  |  | = |  |  |
| Dubuc, J. et al., 2011 | ABs | metritis | No use of AMs | = |  |  |  | = | - |  |
| Elmetwally, M. A. et al., 2020 | ABs | endometritis | Other | - |  |  |  |  |  |  |
| Elsener, J. et al., 2001 | AHs | Gastrointestinal nematode infections | No use of AMs | - |  |  |  | - |  |  |
| Fuenzalida, M. J. and Ruegg, P. L., 2019 | ABs | Clinical mastitis | No use of AMs | + |  |  |  | = | = | = |
| Fuenzalida, M. J. and Ruegg, P. L., 2019 | ABs | Clinical mastitis | No use of AMs |  |  |  |  | = | = |  |
| Galvao, K. N. et al., 2009 | ABs | endometritis (clinical and subclinical) | No use of AMs | = |  |  |  |  | - |  |
| Galvao, K. N. et al., 2020 | ABs | metritis | Prebiotic |  |  |  |  | - |  |  |
| Giuliodori, M. J. et al., 2013 | ABs | metritis | No use of AMs | - |  |  |  | = |  |  |
| Godden, S. et al., 2003 | ABs | Mastitis | New therapy protocol |  |  |  |  | + | + |  |
| Gomez, D. E. et al., 2017 | ABs | diarrhoea | New therapy protocol |  | + |  |  | = | = |  |
| Gomez, D. E. et al., 2021 | ABs | diarrhoea | Animal Health Advisory/Training |  | + |  |  | = | = |  |
| Goshen, T. and Shpigel, N. Y.., 2006 | ABs | clinical metritis and retained placenta | No use of AMs | - |  |  |  |  |  |  |
| Griffioen, K. et al., 2021 | ABs | mastitis (clinical and subclinical) | New therapy protocol |  | - |  |  | = | = | = |
| Guccione, J. et al., 2014 | ABs | mastitis | No use of AMs | - |  | - |  | - |  | - |
| Guccione, J. et al., 2020 | ABs | mastitis (clinical and subclinical) | No use of AMs | = |  |  |  |  | - | - |
| Hallberg, J. W. et al., 2006 | ABs | mastitis (clinical and subclinical) | No use of AMs |  |  |  |  | - | - |  |
| Hektoen, L. et al., 2004 | ABs | mastitis (clinical and subclinical) | Other |  |  |  |  | = |  |  |
| Hendrick, S. H. et al., 2006 | ABs | Johne's disease | No use of AMs |  |  |  |  | - |  |  |
| Jacobs, C. et al., 2018 | ABs | digital dermatitis | Other |  |  |  |  | = | = |  |
| Jeremejeva, J. et al., 2012 | ABs | acute puerperal metritis (APM) and clinical metritis (CM) | New therapy protocol | = |  |  |  | = |  |  |
| Joachim, A. et al., 2003 | ABs | cryptosporidiosis | Other |  |  |  |  | + |  |  |
| Kabera, F. et al., 2020 | ABs | mastitis | New therapy protocol | = |  |  |  |  | = | = |
| Kai, K. et al., 2002 | ABs | mastitis | Bioactive Protein And Peptides |  |  |  |  | + |  |  |
| Kaithwas, G. et al., 2011 | ABs | mastitis | Plant-Based (Phytogenic) |  |  |  |  | = |  | = |
| Kaneene, J. B. et al., 2008 | ABs | diarrhea | Feed/Diet Management |  |  |  | + |  | = |  |
| Kasimanickam, R. et al., 2005 | ABs | subclinical endometritis | Other | = |  |  |  |  |  |  |
| Kasravi, R. et al., 2011 | ABs | sub-clinical mastitis | No use of AMs |  |  |  |  | - | = | - |
| Keller, D. and Sundrum, A., 2018 | ABs | mastitis | Other |  |  |  |  | - |  | - |
| Knutti, B. et al., 2000 | ABs | endometritis | Other | - |  |  |  |  |  |  |
| Kreiger, M. et al., 2007 | ABs | mastitis | No use of AMs | - |  |  |  | - |  | = |
| Kuipers, A. et al., 2016 | ABs | no info | Animal Health Advisory/Training |  | + |  |  |  |  |  |
| Lago, A. et al., 2011 | ABs | Clinical mastitis | New therapy protocol | + | + |  |  | = | = |  |
| Lago, A. et al., 2011 | ABs | Clinical mastitis | New therapy protocol | = | = |  |  | = | = | = |
| Laven, R. A. and Hunt, H., 2002 | ABs | digital dermatitis | Farm Management |  |  |  |  | = |  |  |
| LeBlanc, S. J. et al., 2002 | ABs | Clinical Endometritis | Other | - |  |  |  | = |  |  |
| Leitner, G. et al., 2018 | ABs | mastitis (clinical and subclinical) | Other | + |  |  |  |  | + | + |
| Lendzele, S. S. et al., 2021 | ABs | FMD | Other | = |  | = |  | + |  |  |
| Machado, V. S. and Bicalho, R. C., 2018 | ABs | mastitis (clinical and subclinical) | New therapy protocol | = |  |  |  |  | - | - |
| Madoz, L. V. et al., 2017 | ABs | clinical endometritis | New therapy protocol | = | + |  |  | = |  |  |
| Mahendran, S. A. et al., 2017 | ABs | pneumonia | New therapy protocol | = | + |  |  | - |  |  |
| Malinowski, E. et al., 2019 | ABs | clinical mastitis | New therapy protocol |  |  |  |  | + |  |  |
| Manske, T. et al., 2002 | ABs | digital dermatitis | Farm Management |  |  |  |  | - |  |  |
| Mari, G. et al., 2012 | ABs | endometritis | No use of AMs |  |  |  |  | - |  |  |
| McDougall, S. et al., 2013 | ABs | postpartum endometritis | Other | = |  |  |  | = |  |  |
| McDougall, S. et al., 2022 | ABs | Clinical mastitis | New therapy protocol |  | - |  |  | + | + |  |
| Mullen, K. A. et al., 2014 | ABs | mastitis | Plant-Based (Phytogenic) | = |  |  |  | = | = | = |
| Orjales, I. et al., 2016 | ABs | mastitis | No use of AMs |  |  |  |  |  |  | - |
| Passchyn, P. et al., 2013 | ABs | mastitis | No use of AMs | = |  |  |  |  | <> | = |
| Pempek, J. A. et al., 2019 | ABs | Diarrhea | Bioactive Protein And Peptides |  | = |  |  |  | = |  |
| Pereira, R. V. et al., 2020 | ABs | bovine respiratory disease (BRD) | No use of AMs |  |  |  | + | = |  |  |
| Pinedo, P. J. et al., 2015 | No AMs used | toxic puerperal metritis (TPM) | Other | + |  |  |  | + |  |  |
| Rajala-Schultz, P. J. et al., 2011 | ABs | mastitis | New therapy protocol | <> |  |  |  |  |  | <> |
| Raymond, M. J. et al., 2006 | ABs | Multiple | Animal Health Advisory/Training |  | + |  |  |  |  |  |
| Relun, A. et al., 2013 | Other | digital dermatitis | Farm Management |  |  |  |  |  | = |  |
| Rowe, S. M. et al., 2020 | ABs | mastitis | New therapy protocol |  | + |  |  |  | = |  |
| Rowe, S. M. et al., 2020 | ABs | mastitis | New therapy protocol | = |  |  |  |  | = | = |
| Roy, J. et al., 2007 | ABs | mastitis | No use of AMs | - |  |  |  | - | - | = |
| Roy, J. et al., 2009 | ABs | mastitis | No use of AMs |  |  |  |  | - |  |  |
| Runciman, D. J. et al., 2008 | ABs | endometritis | No use of AMs | - |  |  |  |  |  |  |
| Salat, O. et al., 2008 | ABs | subclinical mastitis | No use of AMs |  |  |  |  | - |  | - |
| Sampimon, O. C. et al., 2009 | ABs | clinical mastitis | No use of AMs | - |  |  |  |  | - | - |
| Sandgren, C. H. et al., 2008 | ABs | subclinical mastitis | New therapy protocol | = |  |  |  | = | = | - |
| Sannmann, I. et al., 2013 | ABs | puerperal metritis | New therapy protocol | = |  |  |  | = | = |  |
| Santman-Berends, I. M. G. A. et al., 2021 | ABs | mastitis | Regulation |  | + |  |  |  |  | - |
| Schmenger, A. et al., 2020 | ABs | clinical mastitis | New therapy protocol |  | + |  |  | = | = |  |
| Schultz, N. and Capion, N., 2013 | ABs | digital dermatitis | New therapy protocol |  |  |  |  | = |  |  |
| Sellera, F. P. et al., 2021 | ABs | digital dermatitis | Other |  |  |  |  | + | + |  |
| Shearer, J. K. and Hernandez, J.., 2000 | ABs | digital dermatitis | Other |  |  |  |  | + |  |  |
| Shephard, R. W. et al., 2000 | ABs | mastitis | No use of AMs | + |  |  |  | = |  |  |
| Shim, E. H. et al., 2004 | ABs | clinical mastitis | New therapy protocol | - |  | - |  |  |  |  |
| Shinozuka, Y. et al., 2009 | ABs | clinical mastitis | Other | + |  |  |  | + |  |  |
| Silva, L. A. F. et al, 2005 | ABs | digital dermatitis | Multiple |  |  |  |  | - |  |  |
| Speksnijder, D. C. et al., 2017 | ABs | no info | Animal Health Advisory/Training | = | = |  |  |  | = | = |
| St Rose, S. G. et al., 2003 | ABs | subclinical mastitis | No use of AMs | = |  |  |  | - |  | - |
| Steele, N. and McDougall, S., 2014 | ABs | subclinical mastitis | No use of AMs |  |  |  |  | - | - | - |
| Stevens, M. et al., 2019 | No AMs used | mastitis | Animal Health Advisory/Training |  | + |  |  | + |  | + |
| Suojala, L. et al., 2010 | ABs | clinical mastitis | New therapy protocol | = |  |  |  | = |  |  |
| Tartor, Y. H. et al., 2020 | AFs | Dermatophytosis | Plant-Based |  |  |  |  | = |  |  |
| Taurel, A. et al., 2012 | ABs | Q fever | Vaccines |  |  |  |  | + | - |  |
| Teixeira, A. G. V. et al., 2017 | ABs | bovine respiratory disease (BRD) | No use of AMs | = |  |  |  |  | - |  |
| Tetens, J. L. et al., 2019 | ABs | no info | New therapy protocol |  |  |  | + |  |  |  |
| Tison, N. et al., 2017 | ABs | clinical endometritis | No use of AMs | - |  |  |  | - |  |  |
| Tomazi, T. et al., 2021 | ABs | clinical mastitis | No use of AMs |  |  |  |  | - |  | - |
| van den Borne, B. H. P. et al., 2019 | ABs | mastitis (clinical and subclinical) | No use of AMs | = |  |  |  |  | = | - |
| Vasquez, A. K. et al., 2015 | ABs | clinical mastitis | New therapy protocol | = |  |  |  | = |  | = |
| Vasquez, A. K. et al., 2018 | ABs | clinical mastitis | New therapy protocol | = |  |  |  | - | = | = |
| Werner, C. et al., 2010 | ABs | clinical mastitis | Other |  |  |  |  | = |  |  |
| Whist, A. C. et al., 2006 | ABs | clinical mastitis | New therapy protocol |  |  |  |  |  | + |  |
| Wittek, T. et al., 2018 | ABs | mastitis | No use of AMs | - |  |  |  |  | = | - |
| Woolford, M. W. et al., 2001 | ABs | clinical mastitis | New therapy protocol |  |  |  |  |  | - |  |
| Zobel, R. and Tkalcic, S., 2013 | ABs | Clinical Endometritis | Other | = |  |  |  | = | = |  |
| Goats |  |  |  |  |  |  |  |  |  |  |
| McDougall, S. et al., 2010 | ABs | subclinical mastitis | No use of AMs | = |  | + |  | - |  | = |
| Layers |  |  |  |  |  |  |  |  |  |  |
| Caudell, M. A. et al., 2022 | ABs | no info | Animal Health Advisory/Training |  | + |  |  |  |  |  |
| Ramirez, S. Y. et al., 2021 | ABs | no info | Plant-Based (Phytogenic) | + |  |  |  |  | = | + |
| Schwaiger, K. et al., 2010 | ABs | no info | Farm Management |  |  |  | + |  | = |  |
| Multiple |  |  |  |  |  |  |  |  |  |  |
| Berge, A. C. B. et al., 2009a | ABs | Multiple | Feed/Diet Management | + |  |  |  |  | + |  |
| Jabbar, A. et al., 2022 | AHs | Gastrointestinal nematode infections | Plant-Based |  |  |  |  | = |  |  |
| Kaiaty, A. M. et al., 2021 | AHs | Gastrointestinal nematode infections | Plant-Based |  |  |  |  | = |  |  |
| Lhermie, G. et al., 2017 | ABs | bovine respiratory disease (BRD) | No use of AMs |  |  |  | = | = |  |  |
| Sheep |  |  |  |  |  |  |  |  |  |  |
| Astobiza, I. et al., 2013 | ABs | Q fever | Multiple |  |  |  |  | = |  |  |
| Croft, A. et al., 2000 | ABs | Clinical mastitis | No use of AMs | - |  |  |  | - | = |  |
| Duncan, J. S. et al., 2012 | ABs | Footrot and contagious ovine digital dermatitis (CODD) | Vaccines |  |  |  |  | + | + |  |
| Gonzalo, C. et al., 2004 | ABs | Mastitis | No use of AMs | = |  |  |  | = | = |  |
| Hernandez, F. et al., 2015 | ABs | mastitis | No use of AMs | - |  |  |  |  |  | - |
| Holsback, L. et al., 2016 | AHs | Gastrointestinal nematode infections | No use of AMs |  |  |  |  | - |  |  |
| Kaler, J. et al., 2010 | ABs | footrot | Multiple |  |  |  |  | - |  |  |
| Learmount, J. et al., 2015 | AHs | Gastrointestinal nematode infections | New therapy protocol |  | + |  | + |  |  |  |
| Learmount, J. et al., 2016 | AHs | Gastrointestinal nematode infections | New therapy protocol | + | + |  |  | = |  |  |
| Maingi, N. et al., 2002 | AHs | Gastrointestinal nematode infections | No use of AMs | - |  | - |  | - |  |  |
| Mugnaini, L. et al., 2013 | No AMs used | dermatophytosis | Plant-Based (Phytogenic) |  |  |  |  | = |  |  |
| Rizzon Cintra, M. C. et al., 2019 | AHs | Gastrointestinal nematode infections | New therapy protocol | = | + |  |  | - |  |  |
| Swine |  |  |  |  |  |  |  |  |  |  |
| Abubakar, R. H. et al., 2019 | ABs | no info | No use of AMs |  |  |  | - |  |  |  |
| Alexopoulos, C. et al., 2006 | ABs | Proliferative enteropathy | No use of AMs | - |  |  |  |  | - |  |
| Arkfeld, E. K. et al., 2015 | ABs |  | No use of AMs | - |  |  |  |  |  | + |
| Byra, C. et al., 2011 | ABs | Streptococcosis | No Alternatives Used |  | = |  |  | - | - |  |
| Cameron-Veas, K. et al., 2016 | ABs | Post-weaning diarrhea | No Alternatives Used |  |  |  | + | + |  |  |
| Che, T. M. et al., 2012 | ABs | no info | Feed/Diet Management | = | = |  |  |  | + |  |
| Chekabab, S. M. et al., 2020 | ABs | Multiple | No use of AMs |  |  |  | + |  |  |  |
| Choi, SH. and Kang, SS., 2001 | ABs | Postpartum hypogalactia syndrome | Other |  |  |  |  | + |  |  |
| Collineau, L. et al., 2017 | ABs | no info | Animal Health Advisory/Training | + | + | <> |  |  | = |  |
| Correa-Fiz, F. et al., 2019 | ABs | no info | No use of AMs | + |  |  |  |  |  |  |
| Correa-Fiz, F. et al., 2020 | ABs | Post-weaning diarrhea | Other | = |  |  |  | = | = |  |
| Cremonesi, P. et al., 2022 | ABs | Post-weaning diarrhea | Other | = |  |  |  |  | = |  |
| De Lucia, A. et al., 2021 | ABs | Colibacillosis | No use of AMs |  |  |  | + |  |  |  |
| Diana, A. et al., 2017 | ABs | no info | No use of AMs | - | - |  |  | <> | = |  |
| Diana, A. et al., 2019 | ABs | no info | Regulation | - | - |  |  | <> | = |  |
| Dohmen, W. et al., 2017 | ABs | no info | Animal Health Advisory/Training |  | + |  |  |  | + |  |
| Dorado-García, A. et al., 2015 | ABs | no info | Animal Health Advisory/Training |  |  |  |  |  | = |  |
| Duggett, N. A. et al., 2018 | ABs | diahorrea | No use of AMs |  |  |  | + |  |  |  |
| Funk, J. et al., 2007 | ABs | Salmonelosis | Farm Management |  |  |  | = |  | + |  |
| Giannakopoulos, C. G. et al., 2001 | ABs | Multiple | No use of AMs | - |  |  |  |  | - |  |
| Heller, O. et al., 2016 | ABs | no info | No use of AMs |  |  |  | + |  |  |  |
| Jensen, K. J. et al., 2022 | ABs | no info | Vaccines |  | = |  |  |  | = |  |
| Kaiser, M. et al., 2013 | ABs | shoulder ulcer | Other |  |  |  |  | + |  |  |
| Kyriakis, S. C. et al., 2002 | ABs | Proliferative enteropathy | No use of AMs | - |  |  |  | - | = |  |
| Kyriakis, S. C. et al., 2002 | ABs | Proliferative enteropathy | No use of AMs | - |  |  |  | - | = |  |
| Laine, T. et al., 2004 | ABs | post-weaning diarrhoea | Regulation | = | = |  |  |  |  |  |
| Mateusen, B. et al., 2001 | ABs | chronic respiratory disease | Vaccines | + | + | - |  | = | = |  |
| Mateusen, B. et al., 2002 | ABs | chronic respiratory disease | Vaccines | = | + |  |  | = | = |  |
| Menezes, T. A. et al., 2020 | ABs | Post-weaning diarrhoea (PWD) | No use of AMs |  |  |  |  | - |  |  |
| Peng, Z. et al., 2016 | ABs | no info | Bioactive Protein And Peptides | + |  |  |  |  | + |  |
| Postma, M. et al., 2017 | ABs |  | Animal Health Advisory/Training | + | + |  |  |  | + |  |
| Raasch, S. et al., 2020 | ABs | no info | Animal Health Advisory/Training |  | + |  |  |  | <> |  |
| Rozeboom, D. W. et al., 2005 | ABs | no info | Prebiotic | = |  |  |  |  | = |  |
| Shen, L. et al., 2021 | ABs | Porcine pleuropneumonia | Animal Health Advisory/Training |  | = |  |  |  |  |  |
| Sjolund, M. and Wallgren, P., 2010 | ABs | no info | Vaccines | = | = |  |  |  | = |  |
| Toya, R. et al., 2022 | ABs | no info | Animal Health Advisory/Training |  | + |  |  |  |  |  |
| van Asseldonk, M. et al., 2020 | ABs | Multiple | Regulation | = | + | = |  |  |  |  |
| Vigre, H. et al., 2008 | ABs | Multiple | Regulation |  |  |  |  | - |  |  |
| Yun, J. et al., 2017 | ABs | no info | No use of AMs | - |  |  | = |  | - |  |
| Tilapia |  |  |  |  |  |  |  |  |  |  |
| Monteiro, S. H. et al., 2016 | ABs | no info | Farm Management |  |  |  | + |  |  |  |
| Turkey |  |  |  |  |  |  |  |  |  |  |
| Van Droogenbroeck, C. et al., 2011 | No AMs used | Respiratory disease | Bioactive Protein and Peptides |  | + | + |  | + | + |  |

# Data classification and management

Table S.4. Class of antibiotics used across the different references and their priority classification according to WHO, WOAH and One Health

| Class of Antibiotic | Priority categorisation | | |
| --- | --- | --- | --- |
|  | **WHO^α1^** | **WOAH^β2^** | **One Healthγ^θ3^** |
| Aminocoumarin | Not used in Human medicine | Important | No overlap |
| Aminocyclitol | Important | Critically important | No overlap |
| Aminoglycosides | CI^#^ - High priority | Highly important | Overlap |
| Aminoglycosides + 2 Deoxystreptamin | CI - High priority | Highly important | Overlap |
| Aminopenicillin + Betalactamase Inhibitor | CI - High priority | Critically important | Overlap |
| Aminopenicillins | CI - High priority | Critically important | Overlap |
| Amphenicols | Highly important | Critically important | No overlap |
| Ansamycin – Rifamycins | CI - High priority | Critically important | Overlap |
| Antistaphylococcal Penicillins | Highly important | Critically important | No overlap |
| Bicyclomycin | Not used in Human medicine | Important | No overlap |
| Cephalosporins First Generation | CI - High priority | Highly important | Overlap |
| Cephalosporins Third Generation | CI - Highest priority | Critically important | Overlap |
| Cephalosporins Fourth Generation | CI - Highest priority | Critically important | Overlap |
| Diaminopyrimidines | CI - High priority | Critically important | Overlap |
| Ionophores | Not used in Human medicine | Highly important | No overlap |
| Lincosamides | Highly important | Highly important | No overlap |
| Macrolides 14- Membered Ring | CI - Highest priority | Critically important | Overlap |
| Macrolides 15- Membered Ring | CI - Highest priority | Critically important | Overlap |
| Macrolides 16- Membered Ring | CI - Highest priority | Critically important | Overlap |
| Natural Penicillins (eg. Benethamine penicillin,  Benzylpenicillin, Penethamate) | Highly important | Critically important | No overlap |
| Orthosomycins | Not used in Human medicine | Important | No overlap |
| Polymyxins (eg. Polymixin B, colistin) | CI - Highest priority | Highly important | Overlap |
| Polypeptides (eg. Bacitracin, Enramycin,  Gramicidin) | Important | Highly important | No overlap |
| Quinolones Second Generation (Fluoroquinolones) | CI - Highest priority | Critically important | Overlap |
| Quinoxalines | Not used in Human medicine | Important | No overlap |
| Streptogramins | Highly important | Important | No overlap |
| Sulfonamides | Highly important | Critically important | No overlap |
| Sulfonamides+ Diaminopyrimidines | Highly important | Critically important | No overlap |
| Tetracyclines | Highly important | Critically important | No overlap |
| *^α^* World Health Organisation^1^; *^β^* World Organisation for Animal Health^2^; *^θ^* according to Venkateswaran, V. *et al*. (2022) ^3^ (unpublished work); ^#^ Critically important; | | | |

# Distribution of antibiotics according to class and importance category


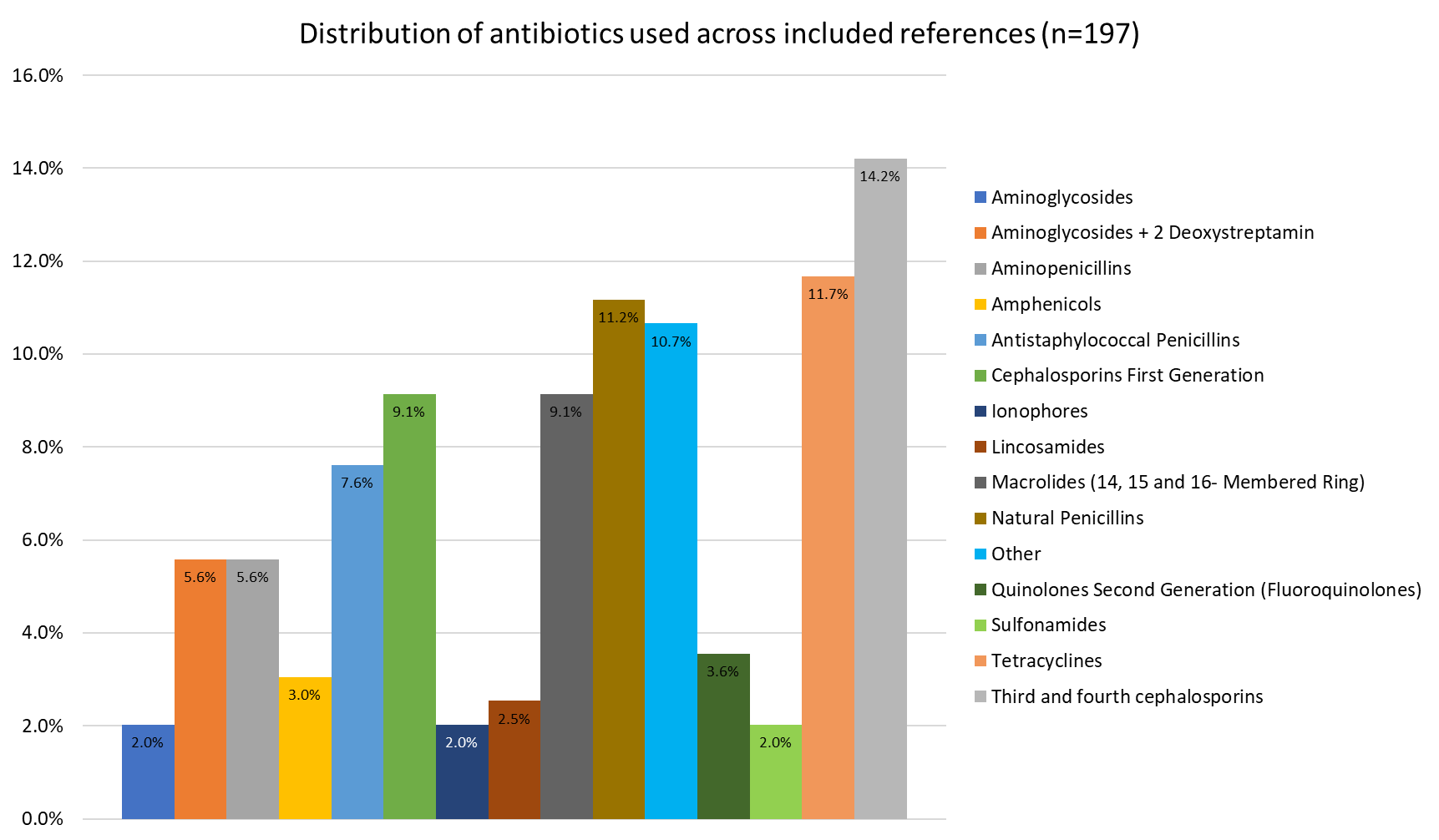


Figure S.1. Relative distribution of the different known antibiotics studied across the references included (note: the n is the number of studies using each of the known antibiotic classes, thus the total number of studies is not equal to n as there could papers that are using am antimicrobial other than antibiotics or not reporting the name of the antibiotic used, or papers that could be using ABs from more than one class)


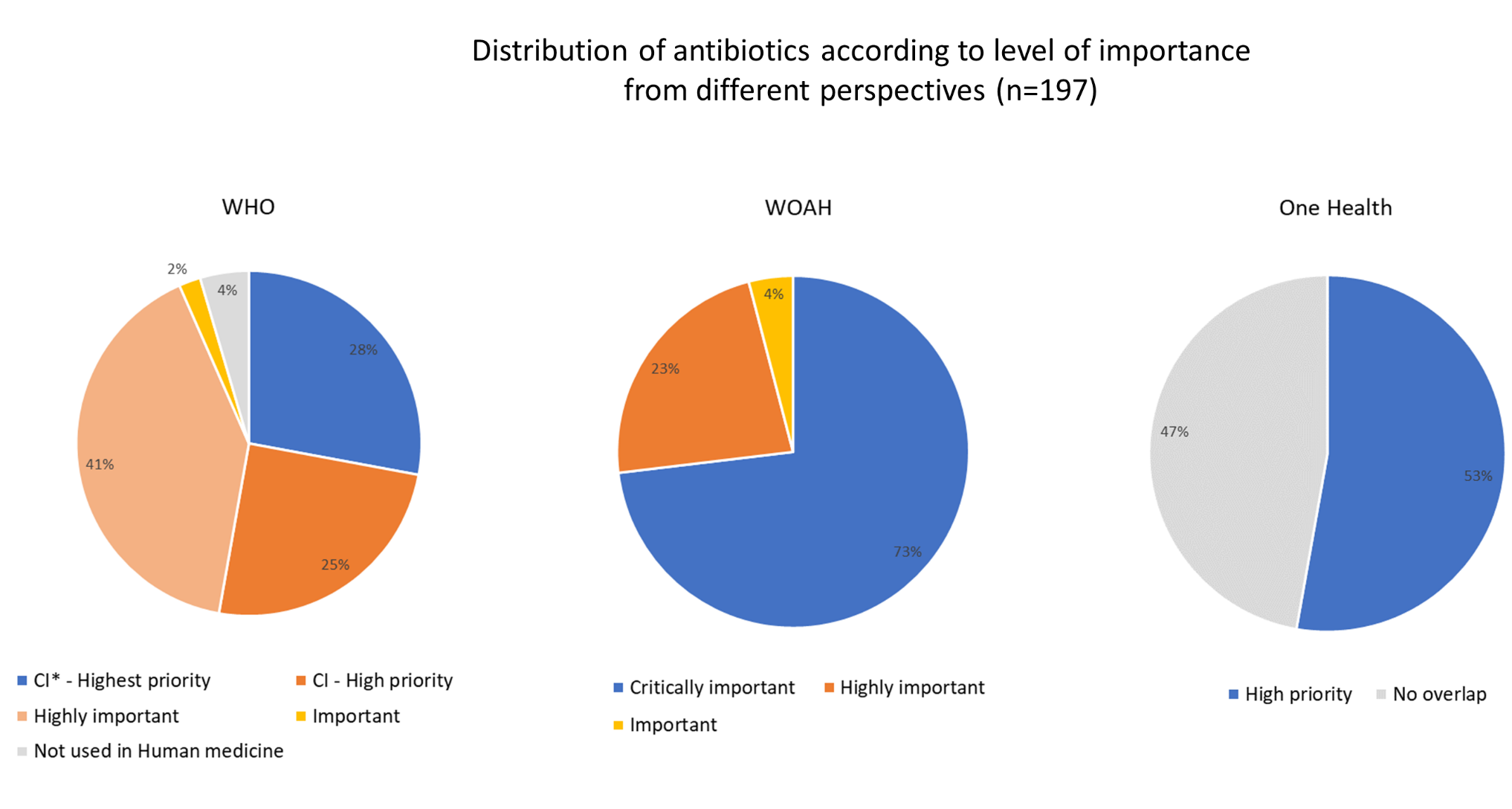


Figure S.2. Relative distribution of the different known antibiotics studied across the references included according to level of importance/priority from a WHO, WOAH and One Health perspective (note: the n is the number of studies using each of the known antibiotic classes, thus the total number of studies is not equal to n as there could papers that are using antimicrobial other than antibiotics or not reporting the name of the antibiotic used, or papers that could be using ABs from more than one class) *Critically Important

# Impact of alternatives/Interventions

Table S.5. Distribution of impact of alternatives/interventions according to direction of effect

| According to | | n | Direction of Effect | | | |
| --- | --- | --- | --- | --- | --- | --- |
|  |  |  | Bidirectional | Negative | Equivalent | Positive |
| Alternatives/Interventions - Overall | | | | | | |
|  | All | 485 | 1.9% | 28.5% | 43.5% | 26.2% |
|  | Excluding *No use of AMs* category | 328 | 2.1% | 13.7% | 50.0% | 34.1% |
| Alternatives/Interventions - Specific | | | | | | |
|  | Animal Health Advisory/Training | 36 | 11.1% | 2.8% | 33.3% | 52.8% |
|  | Bioactive Protein and Peptides | 12 | - | - | 33.3% | 66.7% |
|  | Farm Management | 52 | - | 9.6% | 48.1% | 42.3% |
|  | Feed/Water Management | 11 | - | - | 45.5% | 54.5% |
|  | New therapy protocol | 118 | 1.7% | 20.3% | 54.2% | 23.7% |
|  | No use of AMs | 157 | 1.3% | 59.2% | 29.9% | 9.6% |
|  | Other | 30 | - | 10.0% | 53.3% | 36.7% |
|  | Plant-Based (Phytogenic) | 16 | - | 6.3% | 81.3% | 12.5% |
|  | Prebiotics | 13 | - | 30.8% | 61.5% | 7.7% |
|  | Probiotics | 3 | - | 33.3% | 33.3% | 33.3% |
|  | Regulation | 12 | 8.3% | 33.3% | 41.7% | 16.7% |
|  | Vaccines | 25 | - | 8.0% | 44.0% | 48.0% |

Table S.6. Distribution of the impact of the different alternatives/interventions according to the direction of effect across the different species

| Species | Alternative/Intervention | n | Direction of effect (row %) | | | |
| --- | --- | --- | --- | --- | --- | --- |
|  |  |  | Bidirectional | Negative | Equivalent | Positive |
| Beef cattle | |  |  |  |  |  |
|  | Animal Health Advisory/Training | 3 | - | - | 66.7% | 33.3% |
|  | Farm Management | 10 | - | - | 30.0% | 70.0% |
|  | New therapy protocol | 5 | - | 40.0% | 60.0% | - |
|  | No use of AMs | 20 | - | 70.0% | 25.0% | - |
|  | Other | 3 | - | - | 100.0% | - |
|  | Vaccines | 1 | - | - | - | 100.0% |
| Broiler |  |  |  |  |  |  |
|  | Animal Health Advisory/Training | 6 | 33.3% | 16.7% | 16.7% | 33.3% |
|  | Farm Management | 1 | - | - | - | 100.0% |
|  | Feed/Water Management | 4 | - | - | 25.0% | 75.0% |
|  | No use of AMs | 5 | - | 20.0% | 60.0% | 20.0% |
|  | Prebiotics | 4 | - | - | 75.0% | 25.0% |
|  | Probiotics | 2 | - | - | 50.0% | 50.0% |
|  | Vaccines | 5 | - | - | - | 100.0% |
| Dairy cattle | |  |  |  |  |  |
|  | Animal Health Advisory/Training | 12 | - | - | 50.0% | 50.0% |
|  | Bioactive Protein And Peptides | 6 | - | - | 66.7% | 33.3% |
|  | Farm Management | 32 | - | 9.4% | 59.4% | 31.3% |
|  | Feed/Water Management | 2 | - | - | 50.0% | 50.0% |
|  | New therapy protocol | 104 | 1.9% | 19.2% | 56.7% | 22.1% |
|  | No use of AMs | 82 | 1.2% | 63.4% | 30.5% | 4.9% |
|  | Other | 22 | - | 13.6% | 45.5% | 40.9% |
|  | Plant-Based (Phytogenic) | 8 | - | 12.5% | 87.5% | - |
|  | Prebiotics | 4 | - | 100.0% | - | - |
|  | Probiotics | 1 | - | 100.0% | - | - |
|  | Regulation | 2 | - | 50.0% | - | 50.0% |
|  | Vaccines | 2 | - | 50.0% | - | 50.0% |
| Goat |  |  |  |  |  |  |
|  | No use of AMs | 4 | - | 25.0% | 50.0% | 25.0% |
| Layer |  |  |  |  |  |  |
|  | Animal Health Advisory/Training | 1 | - | - | - | 100.0% |
|  | Farm Management | 2 | - | - | 50.0% | 50.0% |
|  | Plant-Based (Phytogenic) | 3 | - | - | 33.3% | 66.7% |
| Multiple | |  |  |  |  |  |
|  | Feed/Water Management | 2 | - | - | - | 100.0% |
|  | No use of AMs | 2 | - | - | 100.0% | - |
|  | Plant-Based (Phytogenic) | 2 | - | - | 100.0% | - |
| Sheep |  |  |  |  |  |  |
|  | Farm Management | 4 | - | 50.0% | 25.0% | 25.0% |
|  | New therapy protocol | 9 | - | 22.2% | 22.2% | 55.6% |
|  | No use of AMs | 13 | - | 61.5% | 38.5% | - |
|  | Plant-Based (Phytogenic) | 1 | - | - | 100.0% | - |
|  | Vaccines | 3 | - | - | 33.3% | 66.7% |
| Swine |  |  |  |  |  |  |
|  | Animal Health Advisory/Training | 14 | 14.3% | - | 21.4% | 64.3% |
|  | Bioactive Protein And Peptides | 2 | - | - | - | 100.0% |
|  | Farm Management | 2 | - | - | 50.0% | 50.0% |
|  | Feed/Water Management | 3 | - | - | 100.0% | - |
|  | No use of AMs | 31 | 3.2% | 54.8% | 16.1% | 25.8% |
|  | Other | 5 | - | - | 60.0% | 40.0% |
|  | Plant-Based (Phytogenic) | 2 | - | - | 100.0% | - |
|  | Prebiotics | 5 | - | - | 100.0% | - |
|  | Regulation | 10 | 10.0% | 30.0% | 50.0% | 10.0% |
|  | Vaccines | 14 | - | 7.1% | 71.4% | 21.4% |
| Tilapia |  |  |  |  |  |  |
|  | Farm Management | 1 | - | - | - | 100.0% |
| Turkey |  |  |  |  |  |  |
|  | Bioactive Protein and Peptides | 4 | - | - | - | 100.0% |

Table S.7. Distribution of the impact of the different alternatives/interventions according to the direction of effect across the different eco-epidemiological outcome groups

| Eco-Epi outcome group | Alternative/Intervention | n | Direction of effect (row %) | | | |
| --- | --- | --- | --- | --- | --- | --- |
|  |  |  | Bidirectional | Negative | Equivalent | Positive |
| AMR | |  |  |  |  |  |
|  | Bioactive Protein and Peptides | 1 | - | - | - | 100.0% |
|  | Farm Management | 5 | - | - | 20.0% | 80.0% |
|  | Feed/Water Management | 1 | - | - | - | 100.0% |
|  | New therapy protocol | 4 | - | - | 25.0% | 75.0% |
|  | No use of AMs | 11 | - | 9.1% | 36.4% | 54.5% |
|  | Prebiotics | 1 | - | - | 100.0% | - |
|  | Probiotics | 1 | - | - | 100.0% | - |
|  | Vaccines | 1 | - | - | - | 100.0% |
| AMU |  |  |  |  |  |  |
|  | Animal Health Advisory/Training | 15 | - | - | 20.0% | 80.0% |
|  | Bioactive Protein and Peptides | 2 | - | - | 50.0% | 50.0% |
|  | Farm Management | 11 | - | 18.2% | 9.1% | 72.7% |
|  | Feed/Water Management | 1 | - | - | - | 100.0% |
|  | New therapy protocol | 14 | - | 14.3% | 7.1% | 78.6% |
|  | No use of AMs | 2 | - | 50.0% | 50.0% | - |
|  | Other | 1 | - | 100.0% | - | - |
|  | Regulation | 4 | - | 25.0% | 25.0% | 50.0% |
|  | Vaccines | 5 | - | - | 40.0% | 60.0% |
| Clinical | |  |  |  |  |  |
|  | Animal Health Advisory/Training | 2 | - | - | 50.0% | 50.0% |
|  | Bioactive Protein and Peptides | 3 | - | - | 33.3% | 66.7% |
|  | Farm Management | 10 | - | 30.0% | 60.0% | 10.0% |
|  | Feed/Water Management | 2 | - | - | 50.0% | 50.0% |
|  | New therapy protocol | 30 | - | 23.3% | 60.0% | 16.7% |
|  | No use of AMs | 41 | 2.4% | 68.3% | 26.8% | 2.4% |
|  | Other | 14 | - | 7.1% | 50.0% | 42.9% |
|  | Plant-Based (Phytogenic) | 7 | - | 14.3% | 85.7% | - |
|  | Prebiotics | 4 | - | 50.0% | 50.0% | - |
|  | Probiotics | 1 | - | 100.0% | - | - |
|  | Regulation | 2 | 50.0% | 50.0% | - | - |
|  | Vaccines | 6 | - | - | 50.0% | 50.0% |
| Economic performance | |  |  |  |  |  |
|  | Animal Health Advisory/Training | 2 | 100.0% | - | - | - |
|  | Bioactive Protein and Peptides | 1 | - | - | - | 100.0% |
|  | Farm Management | 1 | - | - | - | 100.0% |
|  | New therapy protocol | 3 | - | 33.3% | 33.3% | 33.3% |
|  | No use of AMs | 4 | - | 75.0% | - | 25.0% |
|  | Regulation | 1 | - | - | 100.0% | - |
|  | Vaccines | 2 | - | 50.0% | - | 50.0% |
| Epidemiology | |  |  |  |  |  |
|  | Animal Health Advisory/Training | 9 | 11.1% | 11.1% | 55.6% | 22.2% |
|  | Bioactive Protein and Peptides | 3 | - | - | 33.3% | 66.7% |
|  | Farm Management | 13 | - | - | 69.2% | 30.8% |
|  | Feed/Water Management | 4 | - | - | 50.0% | 50.0% |
|  | New therapy protocol | 26 | - | 23.1% | 61.5% | 15.4% |
|  | No use of AMs | 35 | 2.9% | 54.3% | 37.1% | 5.7% |
|  | Other | 6 | - | - | 66.7% | 33.3% |
|  | Plant-Based (Phytogenic) | 3 | - | - | 100.0% | - |
|  | Prebiotics | 4 | - | 25.0% | 50.0% | 25.0% |
|  | Probiotics | 1 | - | - | - | 100.0% |
|  | Regulation | 1 | - | - | 100.0% | - |
|  | Vaccines | 7 | - | 14.3% | 57.1% | 28.6% |
| Product quality | |  |  |  |  |  |
|  | Animal Health Advisory/Training | 2 | - | - | 50.0% | 50.0% |
|  | Bioactive Protein and Peptides | 1 | - | - | 100.0% | - |
|  | Farm Management | 3 | - | - | 100.0% | - |
|  | New therapy protocol | 12 | 8.3% | 25.0% | 66.7% | - |
|  | No use of AMs | 20 | - | 70.0% | 25.0% | 5.0% |
|  | Other | 2 | - | 50.0% | - | 50.0% |
|  | Plant-Based (Phytogenic) | 3 | - | - | 66.7% | 33.3% |
|  | Regulation | 1 | - | 100.0% | - | - |
| Production | |  |  |  |  |  |
|  | Animal Health Advisory/Training | 6 | 16.7% | - | 33.3% | 50.0% |
|  | Bioactive Protein and Peptides | 1 | - | - | - | 100.0% |
|  | Farm Management | 9 | - | - | 55.6% | 44.4% |
|  | Feed/Water Management | 3 | - | - | 66.7% | 33.3% |
|  | New therapy protocol | 29 | 3.4% | 17.2% | 65.5% | 13.8% |
|  | No use of AMs | 44 | - | 61.4% | 29.5% | 9.1% |
|  | Other | 7 | - | - | 71.4% | 28.6% |
|  | Plant-Based (Phytogenic) | 3 | - | - | 66.7% | 33.3% |
|  | Prebiotics | 4 | - | 25.0% | 75.0% | - |
|  | Regulation | 3 | - | 33.3% | 66.7% | - |
|  | Vaccines | 4 | - | - | 50.0% | 50.0% |

# References

1. World Health Organization 2019. *Critically important antimicrobials for human medicine, 6th revision 2018*. <https://iris.who.int/bitstream/handle/10665/312266/9789241515528-eng.pdf>.

2. World Organization for Animal Health 2021. *OIE list of antimicrobial agents of veterinary importance*. <https://www.woah.org/app/uploads/2021/06/a-oie-list-antimicrobials-june2021.pdf>.

3. Venkateswaran N, Swetschinski LR, Fastl C, *et al*. Bacterial Antimicrobial Resistance: Data Gaps and Relationships between Human and Animal Resistance. Available at SSRN: <https://ssrncom/abstract=4346767> In press. 2022.
